# Comprehensive Assessment of Reactogenicity and Safety of the Live-Attenuated Chikungunya Vaccine (IXCHIQ^®^)

**DOI:** 10.3390/vaccines13060576

**Published:** 2025-05-28

**Authors:** Gabriele Maurer, Vera Buerger, Julian Larcher-Senn, Florian Erlsbacher, Stéphanie Meyer, Susanne Eder-Lingelbach, Juan Carlos Jaramillo

**Affiliations:** 1 Valneva Austria GmbH, 1030 Vienna, Austria; gabriele.maurer@valneva.com (G.M.); susanne.eder-lingelbach@valneva.com (S.E.-L.);; 2 Assign Data Management and Statistics GmbH, 6020 Innsbruck, Austria; julian.larcher-senn@assigndmb.com (J.L.-S.); florian.erlsbacher@assigndmb.com (F.E.); 3 Valneva SE, 69002 Lyon, France; stephanie.meyer@valneva.com

**Keywords:** chikungunya, live-attenuated, pooled analysis, safety, subgroup, vaccine

## Abstract

**Background/Objectives:** This overview provides a comprehensive safety evaluation of the approved live-attenuated vaccine VLA1553 (IXCHIQ^®^) for active immunization for the prevention of disease caused by chikungunya virus (CHIKV) in clinical trials. **Methods**: Protocol-defined solicited systemic events (i.e., fever, arthralgia, myalgia, fatigue, and headache) and other unsolicited arthralgia-related events were evaluated. Additionally, during a regulatory review, a broader definition of adverse events of special interest (broad-definition AESIs) (fever and ≥1 AESI symptom within 30 days post-vaccination) was evaluated post hoc. **Results**: The most frequently reported solicited systemic events post-VLA1553 included fever (13.5%), arthralgia (17.2%), myalgia (23.9%), fatigue (28.5%), and headache (31.6%), with very few prolonged symptoms. The incidence of unsolicited arthralgia-related events (arthritis, osteoarthritis, musculoskeletal stiffness, joint stiffness, and joint swelling) was comparable between VLA1553 and placebo groups. Broad-definition AESIs were observed in 11.7% (361/3082) participants (VLA1553) and 0.6% (6/1033) participants (placebo), with a duration of 1–182 days (median: 4 days; prolonged broad-definition AESI [≥1 symptom lasting ≥ 30 days] occurred in 0.5% of participants) (VLA1553) and 4–27 days (median: 8 days) (placebo). Most symptoms contributing to broad-definition AESIs were solicited. In the VLA1553 group, the most common of these symptoms, in addition to fever, were headache (9.1% of participants), fatigue (8.6%), myalgia (7.0%), and arthralgia (5.2%). There were few severe cases (1.6% of participants in the VLA1553 group). **Conclusions**: In clinical trials, VLA1553 showed an acceptable safety profile that was consistent with other live-attenuated vaccines. The incidence of broad-definition AESIs was mainly limited to the immediate post-vaccination period, and broad-definition AESI symptoms were mostly solicited systemic adverse events.

## 1. Introduction

Chikungunya disease is caused by mosquito-borne chikungunya virus (CHIKV), which is widespread in tropical zones, has pandemic potential, and has been identified as a priority pathogen by the Coalition for Epidemic Preparedness Innovation (CEPI) and the United States (US) National Institutes of Allergy and Infectious Disease (NIAID) [1,2,3,4]. CHIKV is transmitted by *Aedes aegypti* and *A. albopictus* mosquitoes, which are becoming more widespread due to increased international travel and globalization, as well as climatic changes that are creating new habitats for these mosquitoes in historically more temperate, non-endemic regions, where chikungunya clusters have been seeded by travelers returning from endemic areas [5,6]. In the UK, CHIKV was the second most common acquired infection in returning travelers over the period 2015–2020 [7], and outbreaks of autochthonous chikungunya have already been reported following the establishment of invasive *Aedes* species [8]. Also, indigenous species of mosquito, such as the European anthrophillic *A. geniculatus*, are additional potential vectors that could further increase the reach of arboviruses such as CHIKV [9].

CHIKV infection is characterized by initial acute symptomatology within 3–7 days after infection [10], typically including fever, rash, and arthralgia, as well as longer-term morbidity including debilitating joint pain that can last several months or even years [1,11,12,13,14]. The severity of CHIKV infection can vary widely, with factors such as the immune response, age, and the presence of pre-existing health conditions playing a crucial role in the clinical course of the infection. In the acute phase, fever is often severe (≥39 °C) and lasts 4–7 days, and arthralgia usually occurs approximately 2–5 days later. During this period, viremia is common and typically resolves within 8 days of the onset of symptoms, and almost 70% of patients develop a macular or papular rash [4]. Almost half of all infected individuals subsequently experience chronic symptomatology, which can occur in all age groups and populations, although neonates, adults with underlying medical conditions, and older adults are particularly at risk of serious complications [1,2,11]. These prolonged, incapacitating rheumatic symptoms, which are characteristic of CHIKV infection, can have a detrimental effect on quality of life, as well as imposing a significant economic burden not only on populations in endemic areas but also on travelers to these regions, resulting in a significant loss of productivity in working-age adults [8,15,16,17,18]. Furthermore, in a population-based cohort study in Brazil, the risk of death was shown to be increased for up to 84 days after the first symptoms [19], and mortality due to CHIKV infection may be under-appreciated, especially in high-risk populations [20,21]. The significant morbidity and mortality associated with CHIKV infection, especially in at-risk populations, coupled with a hospitalization rate of 17%, which can be challenging for healthcare systems during an outbreak [20], and no specific anti-viral treatment or vaccine available at the time, led to the development of a live-attenuated vaccine (VLA1553, IXCHIQ^®^) for active immunization for the prevention of CHIKV disease [22,23,24]. The single-shot vaccine was approved by FDA in November 2023 [25], Health Canada in June 2024 [26], the European Commission in July 2024 [27], and the UK Medicines and Healthcare Products Regulatory Agency in February 2025 [28]. In conjunction with ongoing vector control methods, this vaccine represents a significant development in the control of chikungunya disease [29] and will play a pivotal role in achieving sustainable improvements in global health goals of limiting the expansion of CHIKV circulation [29]. Additionally, VLA1553 has the potential to shape the emergence of other alphaviruses through cross-reactive immunity [30].

The initial clinical development of VLA1553 included a Phase 1 dose-finding trial (VLA1553-101) [31] from which the medium dose level was selected for use in Phase 3 development, a pivotal Phase 3 trial (VLA1553-301) [32], and a lot-to-lot consistency trial (VLA1553-302) [33]. Together, these three trials, all in US adult populations, formed the basis for licensure of VLA1553 since which time longer-term immunogenicity data [34] and interim results from a Phase 3 trial in healthy adolescents (aged 12–17 years) [35] have been reported. Post-licensure requirements included an evaluation of VLA1553 in a pediatric population, and a Phase 2 dose-finding trial (NCT06106581) in children aged 1–11 years is ongoing.

During the initial adult clinical trials, most adverse events (AEs) were acute and generally mild or moderate in severity. From a pooled analysis of safety data [36], episodes of arthralgia were reported more frequently following VLA1553 vaccination than placebo (16.7% and 4.8%, respectively) and adverse events of special interest (AESIs), defined as fever with simultaneous or overlapping symptoms of acute CHIKV infection ([poly]arthralgia/arthritis, back pain and/or neurological symptoms, or macular to maculopapular rash and/or polyadenopathies) with an onset of 2 to 21 days post-vaccination and a duration ≥ 3 days, occurred at a lower incidence (0.3% and 0.1% for VLA1553 and placebo, respectively). To further evaluate symptoms associated with CHIKV infection, AESIs were defined using a broader definition in a post hoc analysis requested by regulators as fever and at least one AESI symptom occurring within 30 days post-vaccination, regardless of the order of their onset and duration and whether or not the symptoms overlapped (broad-definition AESIs, referred to by regulatory bodies using the terminology ‘chikungunya-like adverse reaction’). Although broad-definition AESIs were considered to be related to vaccination, each contributing symptom was assessed for causality by the investigator.

In this overview, we focus on the incidence of specific individual solicited systemic symptoms assessed in the pivotal Phase 3 VLA1553-301 trial (notably fever, arthralgia, myalgia, fatigue, and headache) and those related to arthralgia (arthritis, osteoarthritis, musculoskeletal stiffness, joint stiffness, and joint swelling) that have occurred after VLA1553 vaccination, as well as broad-definition AESIs, as determined in the post hoc analysis. In this way, we provide an in-depth evaluation of the short- and long-term safety of VLA1553 in adults.

## 2. Datasets Included in Overview

Data from the three adult trials that were completed in the USA, a CHIKV non-endemic country, for regulatory submission for licensure, i.e., VLA1553-101, VLA1553-301, and VLA1553-302, are included in this overview of safety. Details of these trials are summarized in Table 1.

The safety analyses for the pivotal Phase 3 trial (VLA1553-301) are described in the IXCHIQ US Prescribing Information [37]. Specific individual systemic symptoms (solicited events of fever, arthralgia, myalgia, fatigue, and headache, and unsolicited events of arthritis, osteoarthritis, musculoskeletal stiffness, joint stiffness, and joint swelling) are described in the following section for the VLA1553-301 trial only.

In addition to the trial protocol-defined safety endpoints, a post hoc analysis included the evaluation of broad-definition AESIs, defined as the occurrence of fever (≥38 °C/≥100.4 °F) and one or more of any of the following: arthralgia or arthritis, myalgia, headache, back pain, rash, lymphadenopathy, or certain neurological, cardiac, or ocular symptoms that started within 30 days after vaccination (regardless of the order of their onset and duration and whether or not symptoms were overlapping). Severe broad-definition AESIs were those that prevented daily activity and/or required medical intervention. The preferred terms that were included for the analysis of broad-definition AESIs are shown in Table 2, and the analysis based on the VLA1553-301 trial is presented in Section 3.1.

Additionally, a pooled analysis of safety data in 3520 participants aged 18 years and above who received VLA1553 in the Phase 1 trial (data from medium dose level only, as this was the dose selected for the Phase 3 trials) and the two initial Phase 3 trials has been reported and published [36]. A similar pooled analysis but with all dose levels from the Phase 1 trial, and also including broad-definition AESIs, is presented in the IXCHIQ^®^ EMA Prescribing Information [38]. This analysis included 3610 participants, and an overview of broad-definition AESIs in this pooled population is provided in Section 3.2 in addition to the analysis for the VLA1553-301 trial in Section 3.1.

## 3. Protocol-Defined Safety Analysis

The data included in this section derive from the VLA1553-301 analysis.

### 3.1. Solicited Systemic Adverse Events

**Figure 1:**
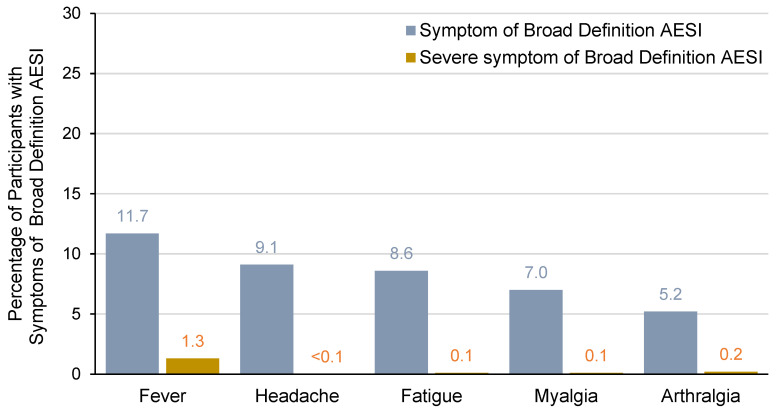
Symptoms of broad-definition AESIs occurring at an incidence of >5% (all severities) for all VLA1553 recipients in VLA1553-301 trial (safety population).

In VLA1553-301, solicited systemic AEs included fever, arthralgia, myalgia, fatigue, headache, nausea/vomiting, and rash. These AEs were collected using an eDiary between Study Day 1 and Study Day 11, and the assessment of severity (i.e., mild, moderate, or severe) was based on FDA Guidance on Toxicity Grading Scales. In the following sections, we focus on the most commonly reported solicited systemic AEs and most commonly reported broad-definition AESI symptoms (i.e., in the 30 days after vaccination) in addition to fever (arthralgia, myalgia, headache, and fatigue) (Figure 1).

#### 3.1.1. Fever

The incidence of solicited fever (≥38.0 °C) was higher in the VLA1553 group (415/3082 participants [13.5%]) than in the placebo group (9/1033 participants [0.9%]). For both VLA1553 and placebo, most episodes of fever were mild in severity (254/415 participants with fever [61.2%] in the VLA1553 group and 6/9 participants with fever [66.7%] in the placebo group) and severe fever (≥39.0 °C) only occurred in the VLA1553 group (44/3082 participants [1.4%]). In the VLA1553 group, the incidence of fever was higher for participants aged 18–27 years (19.8%) than for older age ranges and was similar in participants aged 28–37 years (11.8%), 38–47 years (12.3%), 48–57 years (12.8%), 58–67 years (12.0%), and 68–77 years (12.0%). The incidence was slightly lower in participants aged ≥ 78 years (7.4%); however, the interpretation is limited due to the small sample size (Supplementary Figure S1A). Most episodes of fever in the VLA1553 group were mild in severity for each age group, although a small number of moderate episodes were also reported across age groups except those aged ≥ 78 years, and there were few episodes of severe fever with the incidence declining slightly with age (2.3% of participants aged 18–27 years, 2.1% aged 28–37 years, 1.5% aged 38–47 years, 1.1% aged 48 to 57 years, 0.8% aged 58–67 years, 0.0% aged 68–77 years, and 0.0% aged ≥ 78 years); in the placebo group, the incidence of fever was also consistent across age groups (Supplementary Figure S1A). Overall, solicited fever was reported in both younger and older participants, and there was no indication of an increased incidence of severe fever with age.

The median (minimum, maximum) onset of fever was Study Day 5 (1, 11) for the VLA1553 group and Study Day 4 (1, 11) for the placebo group. The median (minimum, maximum) duration of fever was 2 (1, 94) days for the VLA1553 group and 2 (1, 25) days for the placebo group, and for most of these participants, fever lasted 1 to 5 days (Supplementary Figure S2A). Episodes of fever could have been intermittent, but a conservative approach was used to consider intermittent episodes as a single, longer episode calculated from the start of the first event until the end of the last event. Overall, 415 participants (VLA1553) and 9 participants (placebo) experienced solicited fever, for whom the fever lasted 1 to 5 days for 400 participants (96.4%) and 8 participants (88.9%), respectively. For participants with severe fever (44 participants in the VLA1553 group only) (NB, the entire fever event was marked as severe, even if the severe fever period [i.e., ≥39.0 °C] was shorter), the median (minimum, maximum) duration was 2 (1, 9) days. Most episodes of severe fever lasted 1 to 5 days (40/44 participants [90.9%]) with the remaining 4 participants (9.1%) having severe fevers 7 to 9 days in duration. The median (minimum, maximum) duration of severe fever (if only the period of severe fever was considered, i.e., ≥39.0 °C) was 1 (1, 2) day.

Very few participants had longer-lasting fever (6 to 10 days duration for 12/415 participants with solicited fever [2.9%], 11 to 12 days for 2/415 participants [0.5%] for VLA1553, and 25 days for 1/9 participants [11.1%] for placebo [this episode of fever was coincident with COVID-19 infection]). Only one participant (VLA1553 group), who was lost to follow-up, experienced a fever lasting > 30 days; this estimate of the duration was conservative and based on the confirmed time that the participant was lost to follow-up at Study Day 97 rather than the end of the event.

#### 3.1.2. Arthralgia

The incidence of solicited arthralgia was higher in the VLA1553 group (529/3082 participants [17.2%]) than the placebo group (51/1033 participants [4.9%]). For both the VLA1553 and placebo groups, most episodes of arthralgia were mild in severity (436/529 participants with arthralgia [82.4%] and 49/51 participants with arthralgia [96.1%], respectively). Severe arthralgia only occurred in the VLA1553 group (10/3082 participants [0.3%]). In the VLA1553 group, there was a trend for episodes of arthralgia to decrease with age (20.0% of participants aged 18–27 years, 14.9% aged 28–37 years, 18.8% aged 38–47 years, 17.9% aged 48 to 57 years, 16.1% aged 58–67 years, 12.5% aged 68–77 years, and 11.1% aged ≥ 78 years). Most episodes of arthralgia in the VLA1553 group were mild in severity for each age group, although a small number of moderate episodes were also reported across age groups except those aged ≥ 78 years. There were few episodes of severe arthralgia, with the incidence being consistent across age groups (although the low incidence of fewer than 4 participants [≤0.5%] per age group precluded a robust analysis for severe arthralgia). In the placebo group, the incidence of arthralgia was consistent across age groups (Supplementary Figure S1B). Overall, solicited arthralgia was reported in both younger and older participants, and there was no indication of an increased proportion of arthralgia events with age.

The median (minimum, maximum) onset of arthralgia was Study Day 5 (1, 11) for the VLA1553 group and Study Day 3 (1, 11) for the placebo group. Most episodes of arthralgia were of short duration for both VLA1553 and placebo (Supplementary Figure S2B), and the median (minimum, maximum) duration of arthralgia for VLA1553 and placebo was comparable (2 [1, 182] days [VLA1553] and 3 [1, 180] days [placebo]). Overall, 529 participants (VLA1553) and 51 participants (placebo) experienced solicited arthralgia, for whom the arthralgia lasted 1 to 5 days for 438 participants (83.0%) and 37 participants (72.5%), respectively. There was a total of 13 participants with prolonged solicited arthralgia, i.e., ≥30 days in duration (11/3082 [0.4%] in the VLA1553 group and 2/1033 [0.2%] in the placebo group); additionally, 1 participant had an unsolicited case of prolonged arthralgia, which started 14 days after receiving VLA1553. Of these 14 cases, 5 were classified as broad-definition AESIs and 9 were reported as arthralgia alone: further details are provided in Table 3 (extra information relating to trial completion and other symptoms is included in Supplementary Table S1).

Regarding prolonged arthralgia, 4 participants (3 in the VLA1553 and 1 in the placebo group) experienced arthralgia that lasted approximately 1–2 months (39, 41, 48, and 63 days). Of these, all cases were either recovered (three cases) or recovering/resolving (at the time participant withdrew from the trial [one case]), all were rated by the investigator as mild or moderate in severity, and all were considered by the investigator to be related to VLA1553 or placebo. One of these participants had other prolonged systemic solicited AEs of fatigue, myalgia, and headache, all of which were mild and related. One participant had short-lived systemic solicited AEs of fatigue, fever, and headache, and longer AEs of solicited myalgia and unsolicited back pain, all of which were rated by the investigator as mild and related except for the episode of fever, which was moderate, and this participant had Crohn’s disease (which can have extra-intestinal manifestations, including joint inflammation [39]). One participant had a short-lived systemic solicited AE of myalgia, which was mild and related, and one participant (for whom the arthralgia was reported as right shoulder pain, which is not typical following CHIKV infection) had no other systemic AEs.

Ten participants (nine in the VLA1553 and one in the placebo group) experienced prolonged arthralgia that lasted > 3 months, which was recovered/resolved for three participants, recovering/resolving for two participants (at the time of trial end [one case for VLA1553, one case for placebo]) and not recovered/not resolved (at the time participant was lost to follow-up [one case] or at trial end [four cases]) for five participants (all VLA1553). For the recovered/resolved episodes, all were mild except for one case of severe arthralgia, and all but one (which was reported as right hip pain) were considered to be related. The participant who experienced severe solicited arthralgia, which was likely related to VLA1553, had a relevant medical history of osteoarthritis, which can cause or exacerbate joint pain due to degeneration of cartilage [40]. This participant also had transiently solicited systemic AEs of nausea, fatigue, headache, and a longer-lasting AE of rash (which was not considered to be related to VLA1553). For the recovering/resolving episodes, both were mild or moderate and considered to be related. Both participants had relevant medical histories of spinal stenosis, and one of the participants had additional back pain and neck pain. These participants also had transiently solicited systemic AEs of nausea and fatigue (both participants), and headache or myalgia (each in one participant). For the not recovered/not resolved episodes (ongoing at trial end or lost to follow-up), all were mild or moderate, two were related to VLA1553, and three were not related. For the related episodes, one participant had short-lived solicited systemic AEs of nausea, fatigue, and myalgia, and relevant medical history of osteoarthritis, and the other participant had short-lived solicited systemic AEs of nausea, fatigue, fever, myalgia, and headache. This participant also had a relevant medical history of back pain, foot fracture (which can lead to significant joint pain and discomfort [41]), obesity (which is linked to soft tissue damage and osteoarthritis [42]), and HLA-B27 positivity (a gene allele that is strongly associated with an increased risk of developing certain autoimmune and inflammatory conditions, including arthritis that primarily affects the spine, reactive arthritis, and certain inflammatory bowel diseases [43]). Of the episodes not considered to be related, one was reported as collateral ligament pain in the right knee, one was lost to follow-up so the estimate of duration was conservative (i.e., based on the day the participant was lost to follow-up), and the other was an unsolicited case that started on Study Day 15.

#### 3.1.3. Myalgia

The incidence of solicited myalgia was higher in the VLA1553 group (737/3082 participants [23.9%]) than for placebo (76/1033 participants [7.4%]). In each group, most episodes were mild in severity (617/737 participants with myalgia [83.7%] in the VLA1553 group and 71/76 participants with myalgia [93.4%] in the placebo group). Severe myalgia only occurred in the VLA1553 group (8/3082 participants [0.3%]). In the VLA1553 group, the incidence of myalgia was similar across age groups (25.8% of participants aged 18–27 years, 21.6% aged 28–37 years, 24.9% aged 38–47 years, 24.8% aged 48 to 57, 23.0% aged 58–67 years, 22.6% aged 68–77 years, and 18.5% aged ≥ 78 years), and there were few episodes of severe myalgia (the low incidence of fewer than 3 participants [≤0.4%] per age group precluded a robust analysis for severe myalgia); in the placebo group, the incidence of myalgia was also consistent across age groups (Supplementary Figure S1C).

The median (minimum, maximum) onset of myalgia was Study Day 5 (1, 11) for the VLA1553 group and Study Day 3 (1, 11) for the placebo group. The median (minimum, maximum) duration of myalgia was 2 (1, 194) days for the VLA1553 group and 2 (1, 14) days for the placebo group. Prolonged myalgia (≥30 days) was reported by a total of eight participants (0.3%), all in the VLA1553 group (Table 4 [extra information relating to trial completion and other symptoms is included in Supplementary Table S2]). Of these, three were classified as broad-definition AESIs and five were reported as myalgia alone.

Regarding the prolonged myalgia, four participants experienced myalgia that lasted approximately 1 to 2 months (30, 39, 42, and 48 days), of which two cases were recovered, one case was recovering/resolving (at the time participant withdrew from trial), and one case was not recovered/not resolved (at the time participant withdrew from trial). All cases were considered by the investigator to be related to VLA1553, and all were rated by the investigator as mild in severity. One case, which was not a broad-definition AESI case (absence of fever), was reported as an SAE due to hospitalization and occurred in a 58-year-old female participant with a history of fibromyalgia, back pain, and type 2 diabetes mellitus, who experienced solicited events of mild arthralgia and headache, as well as myalgia, with an onset of symptoms 1 to 2 days after vaccination and unsolicited events of tachycardia and tachypnoea. The participant was hospitalized from Day 4 to Day 10 post-vaccination, and fully recovered, with arthralgia and headache resolving after 1 and 6 days and myalgia resolving after 30 days. Another participant with prolonged myalgia (a duration of 48 days, and non-serious), who also had prolonged arthralgia, had ongoing hypercholesterolemia treated with statins, which have been reported to cause muscle pain [44]. For the other two participants (myalgia duration of 39 days and 42 days), one had ongoing osteoarthritis, the other had no relevant medical history, and both had other systemic solicited AEs of fever, arthralgia, fatigue, or headache, all of which were rated as mild by the investigator except for episodes of moderate arthralgia and headache.

Two participants had prolonged myalgia that lasted approximately 3 months (62 and 75 days). Both cases were recovered/resolved, mild in severity, and considered to be related to VLA1553. One of these participants also had arthralgia, and the other had nausea, fatigue, arthralgia, and headache. All of these solicited systemic events were short-lived, with a duration of 1 to 5 days.

The remaining two participants with prolonged myalgia experienced episodes that lasted 142 and 194 days. For both participants, prolonged myalgia was assessed as mild and not considered by the investigator to be related to VLA1553 vaccination. One participant was obese and had ongoing spinal osteoarthritis, myalgia, and type 2 diabetes mellitus. Increased body fat has been linked to widespread pain, and patients have reported that pain has been exacerbated by obesity [45,46]. Also, spinal osteoarthritis can cause pain and stiffness in the neck and back, which may lead to myalgia due to the stress and strain on surrounding muscles [47], and type 2 diabetes mellitus is significantly associated with myalgia [48]. The second participant also had type 2 diabetes mellitus.

#### 3.1.4. Fatigue

The incidence of solicited fatigue was higher in the VLA1553 group (879/3082 participants [28.5%]) than for placebo (131/1033 participants [12.7%]). In each group, most episodes were mild in severity (783/879 participants with fatigue [89.1%] in the VLA1553 group and 120/131 participants with fatigue [91.6%] in the placebo group). Severe fatigue only occurred in the VLA1553 group (5/3082 participants [0.2%]). In the VLA1553 group, the incidence of fatigue was similar across most age groups (33.5% of participants aged 18–27 years, 31.1% aged 28–37 years, 28.5% aged 38–47 years, 26.1% aged 48 to 57, 25.0% aged 58–67 years, and 27.4% aged 68–77 years) and slightly lower in participants aged ≥ 78 years (14.8%); however, this interpretation is limited due to the small sample size (Supplementary Figure S1D), and fewer than four participants (≤0.5%) experienced severe fatigue in any age group. In the placebo group, the incidence of fatigue was also consistent across most age groups (an increased incidence in participants ≥ 78 years of age was likely due to the small sample size [*N* = 8]) (Supplementary Figure S1D).

The median (minimum, maximum) onset of fatigue was Study Day 4 (1, 11) for the VLA1553 group and Study Day 2 (1, 11) for the placebo group. The median (minimum, maximum) duration of fatigue was 2 (1, 149) days for the VLA1553 group and 2 (1, 184) days for the placebo group.

A total of six participants experienced prolonged solicited fatigue (≥30 days) following VLA1553 vaccination (5/3082 participants [0.2%]) or placebo (1/1033 participants [0.1%]), with onset between the day of vaccination and 4 days post-vaccination. Of these six cases, three were classified as broad-definition AESIs and three were reported as fatigue alone (see below for further details). An additional unsolicited case of prolonged fatigue began 34 days after VLA1553 administration.

Three participants (VLA1553 group) experienced solicited fatigue lasting approximately 1 to 2 months (33, 39, and 69 days), all of which resolved. All cases were considered to be related to VLA1553 and all were mild (two events) or moderate (one event) in severity. One case of prolonged fatigue (33 days) was part of a broad-definition AESI and included events of arthralgia, myalgia, back pain, headache, and lymphadenopathy, with most events resolving within 3 to 7 days (2 events of mild headache persisted for 12 and 15 days, and mild lymphadenopathy for 33 days).

Three participants (two participants in the VLA1553 group and one participant in the placebo group) experienced solicited fatigue lasting ≥ 3 months (94, 149 days [VLA1553] and 184 days [placebo]). In the VLA1553 group, the duration of 94 days was a conservative estimate based on the time that the participant was lost to follow-up since the end date of the event was missing. This event was assessed as mild in severity and unrelated to VLA1553 and was part of a broad-definition AESI with fever and headache that lasted ≥ 30 days (conservative estimate of duration). The other event in the VLA1553 group, with a duration of 149 days, was moderate in severity, deemed related to VLA1553, and resolved. This was part of a broad-definition AESI that included fever, arthralgia, chills, hyperhidrosis, myalgia, headache, and paresthesia, with events lasting 1 to 8 days.

#### 3.1.5. Headache

The incidence of headache was higher in the VLA1553 group (973/3082 participants [31.6%]) than in the placebo group (152/1033 participants [14.7%]). In each group, most episodes were mild in severity (819/973 participants with headache [84.2%] in the VLA1553 group and 134/152 participants with headache [88.2%] in the placebo group). Severe headaches occurred at a low incidence in the VLA1553 group (3/3082 participants [0.1%]) and the placebo group (1/1033 [0.1%]). In the VLA1553 group, the incidence of headache decreased across age groups (42.4% of participants aged 18–27 years, 33.7% aged 28–37 years, 32.9% aged 38–47 years, 28.8% aged 48 to 57, 23.8% aged 58–67 years, 25.0% aged 68–77 years, and 14.8% aged ≥ 78 years), and fewer than 2 participants (≤0.2%) experienced severe headache in any age group; in the placebo group, the incidence of headache also decreased across age groups, with none reported in participants ≥ 78 years of age (although the sample size [*N* = 8] was small in this age group) (Supplementary Figure S1E).

The median (minimum, maximum) onset of headache was Study Day 4 (1, 11) for the VLA1553 group and Study Day 2 (1, 11) for the placebo group. The median (minimum, maximum) duration of headache was 2 (1, 175) days for the VLA1553 group and 2 (1, 77) days for the placebo group.

A total of seven participants experienced prolonged solicited headache (≥30 days) following VLA1553 vaccination (6/3082 participants [0.2%]) or placebo (1/1033 participants [0.1%]) with onset between the day of vaccination and 8 days post-vaccination. Of these seven cases, two were classified as broad-definition AESI and five were reported as headache alone (see below for further details). There were five additional unsolicited cases of prolonged headache (four participants in the VLA1553 group and one participant in the placebo group), which started ≥15 days after vaccination.

Four participants experienced events of solicited headaches lasting approximately 1 to 2 months (30, 33, 39 days [VLA1553], and 77 days [placebo]), all of which resolved. All cases were considered to be related to vaccination, and all were mild (three events) or moderate (one event) in severity. One case (VLA1553 group) of prolonged headache (30 days) was part of a broad-definition AESI and included fever, myalgia, and fatigue, with events resolving within 1 to 7 days.

Three participants (VLA1553 group) experienced solicited headaches lasting > 3 months (94, 165, and 175 days). One VLA1553 recipient, who was lost to follow-up, had mild headaches for 94 days (a conservative estimate based on the time that the participant was lost to follow-up). This event was assessed as unrelated to VLA1553 and was part of a broad-definition AESI with fever and fatigue, lasting ≥ 30 days (conservative estimate of duration). The two other participants had mild headaches for 165 days and 175 days, which were both considered to be related to VLA1553 vaccination (likely since these were initially solicited events) and were ongoing at the end of the trial. One of these participants had a history of migraines and the other had pulmonary hypertension, which was ongoing at study enrolment. Both reported no other AEs such as fever, arthralgia, myalgia, or rash after vaccination.

### 3.2. Unsolicited Arthralgia-Related Adverse Events

Since both acute and chronic arthralgia are characteristic of CHIKV infection, this was included as a solicited AE in the clinical development of VLA1553. However, it is important also to consider other symptoms that are related to arthralgia, including arthritis, osteoarthritis, musculoskeletal stiffness, joint stiffness, and joint swelling, to provide a comprehensive understanding of symptomatology post-VLA1553 vaccination. These symptoms were not solicited, and specific definitions were not used. However, the investigator-documented AEs were coded to the most appropriate MedDRA preferred term, and diagnoses were made per routine clinical care, with the exception of AESI for which the protocol-defined case definition was applied. The data included in this section derive from the VLA1553-301 analysis.

#### 3.2.1. Arthritis

The proportion of participants who experienced unsolicited events of arthritis was the same in the VLA1553 and placebo groups (0.1%), although the incidence was very low in each group (VLA1553: 2/3082 participants; placebo: 1/1033 participants). All events of unsolicited arthritis were considered by the investigator to be unrelated to the trial vaccination.

In the VLA1553 group, the two cases of arthritis started 148 days and 49 days post-vaccination, lasted 12 and 119 days, respectively, and were mild and moderate in severity, respectively. The participant with arthritis that lasted 119 days had elevated rheumatoid factor (which could suggest the presence of an autoimmune disease such as rheumatoid arthritis). Both events of arthritis were non-serious and not medically attended. Neither of these participants reported any arthralgia or fever after the VLA1553 vaccination. The case of arthritis in the placebo group started 21 days post-vaccination and had a duration of 159 days. The events of arthritis with a duration of 119 days (VLA1553) and 159 days (placebo) were reported as ongoing at the end of the trial.

Two other forms of arthritis were reported in separate participants in the VLA1553 group (each reported by 1/3082 participants [0.03%]). These were a case of rheumatoid arthritis and a case of bacterial arthritis, both with onset > 3 months after vaccination (Day 112 and Day 169, respectively). Both events were considered by the investigator to be unrelated to VLA1553 vaccination. Additionally, one participant (0.1%) in the placebo group experienced polyarthritis 14 days post-vaccination.

#### 3.2.2. Osteoarthritis

The proportion of participants who reported osteoarthritis as an unsolicited AE was comparable for VLA1553 (11/3082 participants [0.4%]) and placebo (2/1033 participants [0.2%]). Additionally, a participant (0.03%) in the VLA1553 group experienced an AE of spinal osteoarthritis. All AEs of osteoarthritis were considered by the investigator to be unrelated to VLA1553 vaccination, and most participants (10/12) had a medical history of osteoarthritis, which was ongoing for all of these 10 participants at the time of screening/trial enrolment. No events of osteoarthritis were serious, and none led to discontinuation from the trial.

The participants in the VLA1553 group who had osteoarthritis, including one with spinal osteoarthritis, ranged in age from 38 to 81 years. The majority were White (11 out of 12 participants) and female (8 out of 12 participants), consistent with the higher prevalence of osteoarthritis in women compared to men [49] (Table 5). The majority of participants with osteoarthritis (11 out of 12) were categorized as either overweight (BMI 25.0–29.9 kg/m^2^) or obese (BMI ≥ 30 kg/m^2^), which may be associated with metabolic effects that increase the risk of osteoarthritis in addition to the extra stress on joints due to increased weight [42,49]. Five cases of osteoarthritis in the VLA1553 group worsened within 4 weeks post-vaccination; the remaining 7 cases (including the spinal osteoarthritis) started or worsened later (onset 29–152 days post-vaccination). There was high variation in the duration of the events (range: 1–174 days), and six cases were ongoing at study end (Table 5). Of the five cases (in five participants) that worsened within 4 weeks post-vaccination, two cases (two participants) resolved within 4 days, and two cases (two participants) lasted 19 to 27 days. The remaining participant had worsening chronic pain osteoarthritis that was ongoing at the end of the trial (the duration of 174 days was conservatively calculated based on the end of the trial date). This participant was older (>65 years of age), which can be considered a risk factor for osteoarthritis [49], and had several co-morbidities at screening including diabetes and was obese (BMI ≥ 30 kg/m^2^) (both factors that can increase the risk of osteoarthritis), as well as a history of joint problems/surgical interventions and osteoarthritis. Two participants with osteoarthritis had no reported medical history of osteoarthritis or any other musculoskeletal and connective tissue disorders. In these participants, who both completed the trial, the osteoarthritis occurred at 65 days and 138 days post-VLA1553 vaccination, suggesting no association between VLA1553 vaccination and the onset of osteoarthritis. All events of osteoarthritis in the VLA1553 group were mild in severity, except for two cases that were rated as moderate and had a late onset (148 days and 138 days post-vaccination).

In the placebo group, the two cases (two participants) of osteoarthritis started 26 days and 73 days post-vaccination and were ongoing at the end of the trial, with durations of 158 days and 114 days, respectively (Table 5). Of these, one case was the worsening of osteoarthritis, and the other was reported as osteoarthritis pain to bilateral hands. Both participants had a medical history of osteoarthritis.

#### 3.2.3. Musculoskeletal Stiffness, Joint Stiffness, Joint Swelling

The proportion of participants who experienced unsolicited AEs of musculoskeletal stiffness, joint stiffness, and joint swelling was comparable between the VLA1553 and placebo groups. All were reported with a low frequency (≤0.5%) (musculoskeletal stiffness: 0.4% [VLA1553] and 0.5% [placebo]; joint stiffness: 0.3% and 0.2%; joint swelling: 0.1% and 0.2%). The proportion of participants with events considered to be related to trial vaccination was also comparable between VLA1553 and placebo (0.1% and 0.2%; 0.1% and 0.2%, 0% and 0.1%, respectively). None of the events were serious or medically attended, and all but one (in the placebo group) were mild.

## 4. Broad-Definition Adverse Events of Special Interest

### 4.1. VLA1553-301 Trial (VLA1553 and Placebo)

#### 4.1.1. Overview of Broad-Definition AESIs

The incidence of broad-definition AESIs was higher for VLA1553 (361/3082 participants [11.7%]) than for placebo (6/1033 participants [0.6%]) in the safety population (Table 6). The most common symptoms in addition to fever, occurring at an incidence of >5% in the VLA1553 group, were headache (9.1% of participants), fatigue (8.6%), myalgia (7.0%), and arthralgia (5.2%). Most of these symptoms were solicited (headache [279/282 events, 98.9%]), fatigue [263/265 events, 99.2%], myalgia [214/217 events, 98.6%], and arthralgia [159/162 events, 98.1%]). In the placebo group, the same symptoms in addition to fever were seen in participants with broad-definition AESIs but occurred at lower rates (headache 0.5%, fatigue 0.5%, myalgia 0.1%, and arthralgia 0.2%) (Table 6).

The most common broad-definition AESI symptoms generally overlapped with fever (i.e., the start and/or end of the symptom generally fell within the event duration of fever), including headache in 8.1% of participants in the safety population (89.6% of participants with the broad-definition AESI symptom headache), fatigue in 7.4% (86.7% of participants with the broad-definition AESI symptom fatigue), myalgia in 6.1% (87.4% of participants with the broad-definition AESI symptom myalgia), and arthralgia in 4.4% (84.9% of participants with the broad-definition AESI symptom arthralgia). In the safety population, 5.6% of participants had symptoms of headache and fatigue that overlapped with fever (85.7% of participants with the broad-definition AESI symptoms headache and fatigue). Additionally, 3.4% of participants had symptoms of arthralgia and myalgia that overlapped with fever (83.5% of participants with the broad-definition AESI symptoms arthralgia and myalgia).

Severe symptoms only occurred in the VLA1553 group, and the incidence was low overall (1.6% of participants) and for individual symptoms (fever [1.3% of participants], arthralgia [0.2%], fatigue [0.1%], myalgia [0.1%]) (Table 6 and Figure 1). Single participants (<0.1%) in the VLA1553 group experienced severe symptoms of atrial fibrillation, back pain, and headache (Table 6). The events of severe fever were mostly considered related to VLA1553 vaccination and were generally self-limiting with a duration of 1 to 3 days (only 4/39 participants had fever with a duration between 5 and 8 days). All events of severe arthralgia, myalgia, and fatigue were considered related to VLA1553 vaccination and were self-limiting, lasting 1 to 6 days (arthralgia), 3 to 4 days (myalgia), or 2 to 16 days (fatigue). The single episodes of severe back pain and severe headache were also considered related to VLA1553 vaccination and lasted 50 days and 4 days, respectively (NB, the entire event was marked as severe, even if the severe period was shorter). None of the events of severe fever, arthralgia, myalgia, fatigue, back pain, or headache were serious or needed medical attention. The episode of severe atrial fibrillation required medical intervention but was not considered to be related to VLA1553 vaccination by the investigator. However, a causal relationship could not be entirely ruled out by some regulators.

Other symptoms (all severities) in the VLA1553 group were chills (0.9%), rash (0.7%), back pain (0.4%), lymphadenopathy (0.3%), dizziness (0.2%), pain (0.1%), paresthesia (0.1%), hyperhidrosis (0.1%), peripheral edema (0.1%), and (all <0.1%) asthenia, ataxia, atrial fibrillation, feeling abnormal, hypoesthesia, influenza-like illness, peripheral neuropathy, erythematous rash, and syncope. In the placebo group, the only other symptom was chest pain, which was reported by 0.1% of participants (Table 6).

In the VLA1553 group, the most common combinations of systemic adverse events (which were mostly solicited) that contributed to broad-definition AESI in addition to fever (i.e., headache [9.1%], fatigue [8.6%], myalgia [7.0%], and arthralgia [5.2%]) were also the most commonly reported solicited systemic AEs in VLA1553-301 (31.6%, 28.5%, 23.9%, and 17.2% of participants, respectively) [37]. Broad-definition AESI cases involving other preferred terms generally concerned only smaller numbers (≤1% of participants). The less common symptoms included atrial fibrillation, hypoesthesia, peripheral neuropathy, erythematous rash, influenza-like illness, and syncope that were considered by the investigator to be unrelated to VLA1553, and episodes of mild ataxia, asthenia, and feeling abnormal that were considered to be related to VLA1553 but were short-lived (3 to 8 days).

For the subset of participants in the safety population with broad-definition AESIs (*n* = 361) (Supplementary Figure S3), the broad-definition AESI symptomatology reflected that described above for all participants, albeit with higher incidences due to the smaller sample size of the subset.

A single broad-definition AESI case was serious (the participant was hospitalized), occurring in a 66-year-old male participant who received VLA1553 and experienced fever from 5 to 11 days post-vaccination, which was reported as severe (≥39.0 °C/102.2 °F) for 1 day, and other broad-definition AESI symptoms of mild and moderate fatigue (2 events, onset 6 days and 20 days post-vaccination), moderate myalgia (1 event, onset 3 days post-vaccination), mild headache (2 events, onset 6 and 15 days post-vaccination), and severe atrial fibrillation (1 event, onset 10 days post-vaccination). The participant had a medical history of hypertension, which was ongoing at the time of study enrolment. This participant was hospitalized for 3 days (from 10 to 12 days post-vaccination) due to atrial fibrillation, and also presented with increased troponin, increased brain natriuretic peptide, and hypovolemic hyponatremia. The participant recovered fully.

#### 4.1.2. Broad-Definition AESI in Participants with Underlying Health Conditions

**Figure 2:**
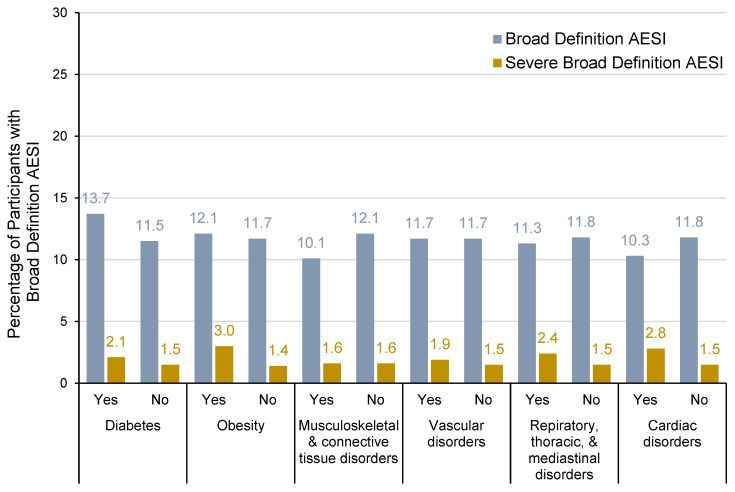
Incidence of broad-definition AESIs for participants with or without type of medical history for VLA1553 in VLA1553-301 trial (safety population). Note: Medical history category ‘diabetes’ included preferred terms (PTs) of type 1 diabetes mellitus, type 2 diabetes mellitus, and diabetes mellitus in the System Organ Class (SOC) metabolism and nutrition disorders; medical history category ‘obesity’ included PT of obesity in the SOC metabolism and nutrition disorders; SOCs of cardiac disorders and musculoskeletal and connective tissue disorders included all PTs for each SOC; SOC of vascular disorders included PTs of hypertension, essential hypertension, and labile hypertension only; SOC of respiratory, thoracic, and mediastinal disorders included PTs of asthma, COPD, exercise-induced asthma, childhood asthma, chronic bronchitis, bronchial hyperactivity, and allergic respiratory disease only. The number of participants (*n*) included in each subgroup was as follows: diabetes (yes, *n* = 233; no, *n* = 2849), obesity, (yes, *n* = 298; no, *n* = 2784), musculoskeletal and connective tissue disorders (yes, *n* = 645; no, *n* = 2437), vascular disorders (yes, *n* = 588; no, *n* = 2494), respiratory, thoracic, and mediastinal disorders (yes, *n* = 248; no, *n* = 2834), cardiac disorders (yes, *n* = 107; no, *n* = 2975).

Individuals with underlying health conditions such as diabetes, hypertension, cardiovascular disease, musculoskeletal disorders, and respiratory disorders are at increased risk of severe complications from acute CHIKV infection, and obesity can further exacerbate the severity of CHIKV symptoms. Furthermore, the systemic inflammation caused by CHIKV can worsen respiratory symptoms and lead to more frequent or severe asthma attacks, while the joint pain and swelling associated with CHIKV infection can aggravate existing musculoskeletal conditions. Based on this, the incidence of broad-definition AESI cases in vaccinated participants with selected medical histories was evaluated. The incidence of broad-definition AESI in the VLA1553 group was consistent among participants with diabetes (13.7% of participants), obesity (12.1%), musculoskeletal and connective tissue disorders (10.1%), vascular disorders (11.7%), respiratory, thoracic, and mediastinal disorders (11.3%), and cardiac disorders (10.3%) (Figure 2). Severe broad-definition AESIs were observed at a low incidence for each medical history (diabetes [2.1% of participants], obesity [3.0%], musculoskeletal and connective tissue disorders [1.6%], vascular disorders [1.9%], respiratory, thoracic, and mediastinal disorders [2.4%], and cardiac disorders [2.8%]). The frequency of broad-definition AESI was similar in participants with or without the evaluated medical histories (Figure 2).

#### 4.1.3. Broad-Definition AESIs by Age Group

**Figure 3:**
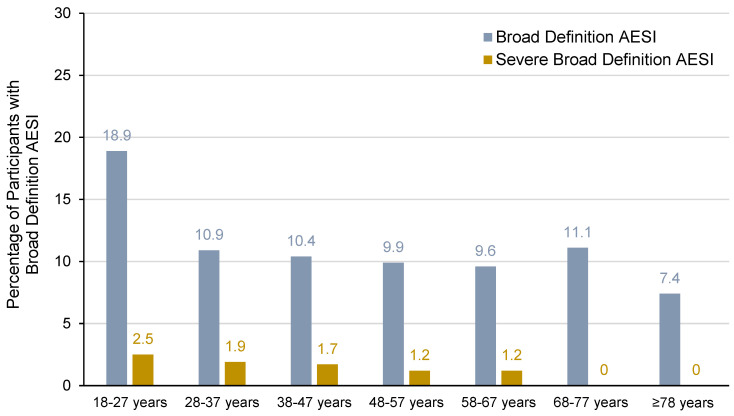
Incidence of broad-definition AESIs by age for VLA1553 in VLA1553-301 trial (safety population). Note: The number of participants (*n*) included in each age group was as follows: 18–27 years (*n* = 519), 28–37 years (*n* = 569), 38–47 years (*n* = 586), 48–57 years (*n* = 664), 58–67 years (*n* = 509), 68–77 years (*n* = 208), ≥78 years (*n* = 27).

Older individuals are at increased risk of severe chikungunya symptoms [50]. In our analysis, the incidence of broad-definition AESI in the VLA1553 group was highest for participants aged 18–27 years (18.9%) compared to the older age ranges and was similar in participants aged 28–37 years (10.9%), 38–47 years (10.4%), 48–57 years (9.9%), 58–67 years (9.6%), and 68–77 years (11.1%). The incidence was slightly lower in participants aged ≥ 78 years (7.4%); however, the interpretation is limited due to the small sample size (see Figure 3). The incidence of severe broad-definition AESIs was low for participants aged 18–27 years (2.5%), 28–37 years (1.9%), 38–47 years (1.7%), 48–57 years (1.2%), and 58–67 years (1.2%) and no severe broad-definition AESIs were observed in participants aged ≥ 68 years.

#### 4.1.4. Onset and Duration of Broad-Definition AESIs

**Figure 4:**
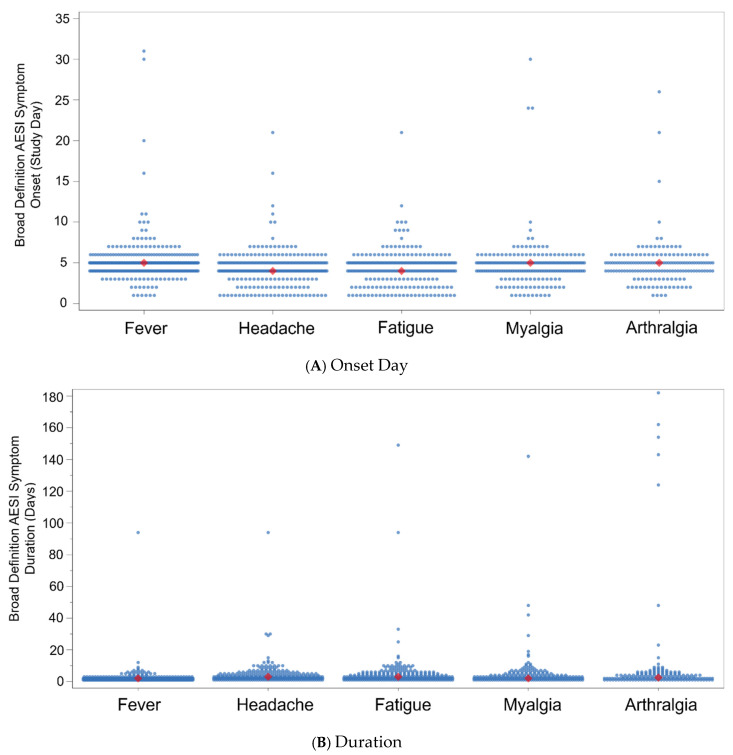
Broad-definition AESI symptoms (>5% frequency) by (**A**) onset day and (**B**) duration for VLA1553 in VLA1553-301 trial (safety population). Note: in Panel (**A**), each dot represents an individual participant’s broad-definition AESI symptom onset day. The diamond-shaped icon indicates the median onset day for each broad-definition AESI symptom type. The median (minimum, maximum) onset for fever, headache, fatigue, myalgia, and arthralgia was Study Day 5 (1, 31), 4 (1, 21), 4 (1, 21), 5 (1, 30), and 5 (1, 26), respectively. In Panel (**B**), ach dot represents an individual participant’s broad-definition AESI symptom duration (days). The diamond-shaped icon indicates the median duration (days) for each broad-definition AESI symptom type. The median (minimum, maximum) duration for fever, headache, fatigue, myalgia, and arthralgia was 2 (1, 94) days, 3 (1, 94) days, 3 (1, 149) days, 2 (1, 142) days, and 2.5 (1, 182) days, respectively.

For broad-definition AESIs, the median (minimum, maximum) onset was Study Day 4 (1, 11) for VLA1553 and Study Day 2.5 (1, 9) for placebo, and the median (minimum, maximum) duration (calculated from the onset of the first symptom until the end of the last symptom) was 4 (1, 182) days for VLA1553 and 8 (4, 27) days for placebo. For individual symptoms that occurred at a frequency of >5%, the median onset for fever, headache, fatigue, myalgia, and arthralgia was Study Days 5, 4, 4, 5, and 5, respectively, and the median duration was 2 days, 3 days, 3 days, 2 days, and 2.5 days, respectively (Figure 4).

Prolonged broad-definition AESIs (i.e., any broad-definition AESI with at least one symptom with a duration ≥ 30 days) were uncommon overall and only occurred in participants who received VLA1553 (21 episodes in 14 participants [0.5% of the safety population and 3.9% of the population of participants who reported broad-definition AESIs]). The most common prolonged broad-definition AESI symptoms were arthralgia (six episodes in five participants), followed by fatigue, headache, and myalgia (each with three episodes in three participants), lymphadenopathy (two episodes in two participants), and back pain, peripheral edema, fever, and erythematous rash (each with one episode in one participant) (Table 7). Further details for prolonged arthralgia and prolonged myalgia are provided in Table 3 (and Supplementary Table S1) and Table 4 (and Supplementary Table S2), respectively. Of these, only one symptom (back pain) was considered by the investigator to be severe (Table 7).

### 4.2. Pooled Results (VLA1553 Only)

For VLA1553, 3610 participants were included in the pooled safety analysis set (i.e., all enrolled participants who were vaccinated at Day 1) (*N* = 3082, *N* = 408, and *N* = 120 in trials VLA1553-301, VLA1553-302, and VLA1553-101, respectively]), and the overall rates of broad-definition AESIs (12.1% of participants), VLA1553-related broad-definition AESIs (11.6%), and severe broad-definition AESIs (1.8%) were consistent with those for the VLA1553-301 trial. As described for VLA1553-301, the incidence of broad-definition AESIs for VLA1553 in the pooled analysis was slightly lower in older than younger participants (although participants aged ≥ 65 years of age were only included in the VLA1553-301 trial) (Table 8). The incidence of broad-definition AESIs was 9.5% for female participants and 15.0% for male participants, which is considered to be comparable with no clinically relevant difference between sexes. For ethnicity, broad-definition AESIs were reported at a similar rate for participants of Hispanic/Latino ethnicity or of non-Hispanic/Latino ethnicity (11.0% and 12.3%, respectively), and differences by race could only be interpreted in a meaningful way for Black/African American (530/3610 participants) and White (2867/3610) participants (all other subgroups were too small). Slightly more broad-definition AESIs were reported in White participants (12.6%) than in Black/African American participants (9.1%), although, again, this difference was not considered to be of clinical importance. The incidence of broad-definition AESIs was consistent when stratified by BMI (12.9%, 12.1%, 10.8%, and 12.6% for BMIs of <25 kg/m^2^, ≥25 kg/m^2^ to <30 kg/m^2^, ≥30 kg/m^2^ to <35 kg/m^2^, and ≥35 kg/m^2^). Overall, the subgroup analysis did not suggest any clinically meaningful differences in the incidence of broad-definition AESIs when stratified by age, sex, ethnicity, race, or BMI.

## 5. Discussion

Although mortality due to CHIKV infection is low, the characteristic acute and chronic morbidity associated with chikungunya disease, particularly long-term arthralgia and related symptoms, has made chikungunya a priority for vaccine development [51]. VLA1553, a live-attenuated vaccine against chikungunya disease, plays a vital role in addressing the related socioeconomic burden of the disease and limiting the global spread of CHIKV. Challenges around its widespread adoption will include education regarding safety and immunogenicity. The recent licensure of VLA1553 (IXCHIQ^®^) has warranted an in-depth evaluation of its safety profile, and the analysis of broad-definition AESIs is critical in this respect.

The assessment of VLA1553 safety has included a consideration of data from the pivotal Phase 3 trial (VLA1553-301) [37], a pooled analysis of all data from the Phase 1 trial (VLA1553-101) and two Phase 3 trials (VLA1553-301 and VLA1553-302) [38], and a pooled analysis of the medium dose data from the Phase 1 trial (i.e., the dose used in later Phase 3 development) together with the two Phase 3 trials [36]. We have further characterized the episodes of solicited fever, arthralgia, myalgia, fatigue, and headache in the VLA1553-301 trial, which all occurred at an incidence of >10% following VLA1553 vaccination and are also seen with other licensed, highly immunogenic vaccines [52,53,54], with a focus on prolonged cases. Solicited systemic AEs occurred to a greater extent after VLA1553 than placebo. Symptoms started within (median) 4 days post-vaccination, were mostly mild in severity, and were usually short-lived with a median duration of 2–3 days; prolonged events (≥30 days) were reported infrequently. Solicited systemic AEs were reported in both younger and older participants and there was no indication of an increased incidence of severe events with age. For symptoms that were considered to be relevant in the evaluation of arthralgia (arthritis, osteoarthritis, musculoskeletal stiffness, joint stiffness, and joint swelling, all of which were not solicited), there was no difference in incidence between VLA1553 vaccination and placebo. In most cases, these were not considered to be vaccine-related, due to their late onset or the presence of other relevant factors, such as underlying medical conditions, and some did not occur in every trial (e.g., no new cases or worsening of existing cases of osteoarthritis were reported in studies VLA1553-101 or VLA1553-302).

Previously, we have reported a low incidence of AESIs, defined as fever (≥38.0 °C/100.4 °F) and at least one other simultaneous or overlapping symptom with onset 2 to 21 days post-vaccination and lasting at least 3 days [36]. Here, we provide an overview of the incidence of AESIs, using a broader and less specific definition (broad-definition AESIs) than the one used for protocol-defined AESIs, which extends to 30 days post-vaccination without any requirement for minimum symptom duration nor for simultaneous or overlapping symptomatology. The broader definition for AESIs provided a more conservative assessment of AESI symptoms following the administration of VLA1553 and was introduced during a regulatory review.

As expected, the incidence of broad-definition AESIs was higher in participants who received VLA1553 (11.7%) than placebo (0.6%). Fever was required for all broad-definition AESI cases, and the most common symptoms contributing to broad-definition AESIs generally coincided with the episode of fever and were commonly reported systemic AEs that are typically collected for vaccine trials. These symptoms were mainly solicited and generally considered to be related to VLA1553 or placebo. Few broad-definition AESI symptoms were severe (1.6% of participants), and these only occurred in the VLA1553 group. The incidence of broad-definition AESIs in older vaccinees was generally comparable to younger adults, with a slight trend toward lower rates as age increased, although overall, age was considered to have no impact on the overall safety profile of VLA1553. This contrasts with CHIKV infection, for which older adults are more at risk of complications than healthy younger adults [1,50]. CHIKV-infected patients with comorbidities such as diabetes or heart disease have shown mortality rates exceeding 10% [55,56], and rates as high as 15% have been reported in elderly patients or those with prior emergency department or intensive care admissions [20]. There was no trend following VLA1553 vaccination in the clinical trials toward a higher incidence of broad-definition AESIs in participants with pre-existing medical conditions that are known to increase the risk for complications from acute CHIKV infection or worsen chikungunya disease symptoms [1,50]. The majority of symptoms contributing to broad-definition AESI occurred soon after vaccination, mostly within the period for collection of solicited adverse events, and very few participants experienced prolonged symptoms contributing to a broad-definition AESI (i.e., ≥1 symptom lasting ≥ 30 days), in contrast to the chronic symptomatology that is characteristic of chikungunya disease [4,57,58]. Only 14 participants (0.5% of the safety population) experienced a prolonged symptom contributing to a broad-definition AESI following VLA1553 vaccination, which included prolonged arthralgia for five participants. A further nine participants experienced prolonged arthralgia that was not identified as a broad-definition AESI case. Importantly, these cases of arthralgia often had other relevant medical conditions that are known to exacerbate joint pain, including Chron’s disease, osteoarthritis, foot fracture, obesity, and HLA-B27 positivity. Additionally, in some cases, the long duration was a conservative estimate due to participant withdrawal, and prolonged cases were also reported in participants who received a placebo.

In a typical presentation of chikungunya, usually with fever and overlapping arthralgia or myalgia, approximately 43% of individuals with acute disease would be expected to progress to chronic symptoms [20], although up to 80% of infected individuals have been reported to display chronic symptoms [4,57,58]. The retrospective broad-definition AESI analysis provided a more conservative analysis than the AESI monitoring that was planned prospectively in the clinical trial protocol, and we observed 11.7% (VLA1553-301 analysis) and 12.1% (pooled analysis) of participants with broad-definition AESIs after VLA1553 vaccination. If this represented chikungunya-associated disease, the resulting hypothetical rate of chronic disease would be 5–10%, whereas, in our analysis, the incidence of prolonged symptoms contributing to broad-definition AESIs was generally <1%. Individuals with chronic chikungunya disease may experience painful, incapacitating symptoms that can last for months or even years, often manifesting as post-chikungunya chronic polyarthralgia or arthritis [55,59]. These chronic symptoms are often compared to rheumatoid arthritis, both in their inflammatory pathogenesis and clinical presentation, including pain and swelling in multiple joints accompanied by stiffness [4,59,60]. For VLA1553, persistent arthralgia (>3 months)—whether reported as a solicited event starting within 10 days after vaccination or as part of broad-definition AESIs—was infrequent (0.3% [9/3082] participants). This is in sharp contrast to the considerably higher proportion of individuals who experience persistent joint pain for more than 3 months following natural CHIKV infection [4,20,57,58] and highlights a key difference in long-term joint-related outcomes between vaccination and natural CHIKV infection. Furthermore, severe solicited arthralgia (onset within 10 days post-vaccination) occurred at a low frequency in VLA1553 recipients (0.3%), and other persistent joint-related complications observed after CHIKV infection, such as arthritis, joint stiffness, musculoskeletal stiffness, and joint swelling, were reported at similar frequencies in the VLA1553 and placebo groups. Together, these findings indicate that VLA1553 was not associated with a higher occurrence of these complications, further supporting its favorable safety profile.

The majority of broad-definition AESIs that we have observed are not considered likely to reflect chikungunya-associated events, being mainly comprised of individual symptoms that are consistent with a strong innate immune response or those generally seen after any vaccination and routinely collected as solicited events [61]. Throughout the clinical development process for VLA1553, an independent data safety monitoring board (DSMB) including clinical experts for chikungunya evaluated vaccine safety. This included an analysis of AESIs and SAEs (low incidences of SAEs [1.5%] and related SAEs [0.1%] were reported in the VLA1553 group in VLA1553-301 [32]), and the DSMB did not identify any significant cause for concern.

A limitation of this analysis is the inclusion of data from only three clinical trials (i.e., the trials that comprised the initial evaluation for licensure). Also, these trials only included adult participants aged ≥ 18 years, and there were few data for older adults aged ≥ 65 years (this population was only available in the pivotal Phase 3 trial), with no data in adolescents or children. Since the licensure of VLA1553 (IXCHIQ^®^), further clinical trials are planned or ongoing in such populations, and continuing post-marketing use and surveillance for safety to date are aligned with the interpretation regarding broad-definition AESIs from the pivotal Phase 3 trial and the pooled analysis for safety. Furthermore, only limited long-term data are included in this analysis, which focuses on an evaluation of safety in the 6 months after vaccination.

## 6. Conclusions

A single dose of VLA1553 showed an acceptable safety profile throughout clinical development that was consistent with other live-attenuated vaccines. The incidence of broad-definition AESIs was mainly limited to the immediate post-vaccination period, and symptoms were mostly solicited systemic adverse events. A range of pooled and subgroup analyses supported the overall safety profile for different age groups and medical histories, with these findings supportive of the licensed VLA1553 vaccine.

## 7. Future Directions

An assessment of antibody persistence in a subset of the VLA1553 cohort from the pivotal Phase 3 trial has been reported up to 2 years after vaccination [34] with ongoing evaluations planned through 10 years post-vaccination. Post-licensure safety monitoring is ongoing, including a post-authorization safety study and a prospective safety cohort study in addition to routine pharmacovigilance activities, to characterize broad-definition AESIs and any other safety concerns that may emerge following the implementation of VLA1553 vaccination programs. Additionally, trials are planned or ongoing in younger age groups (<18 years) and in countries with chikungunya endemicity [35]. In May 2025, following post-marketing reports of SAEs in elderly people with multimorbidities after having received IXCHIQ^®^, FDA paused vaccination in individuals above 60 years of age and EMA temporarily restricted the use of IXCHIQ^®^ in older adults (aged 65 years and above) until investigation of the events has been concluded. For safety updates, refer to the company’s newsroom at https://valneva.com/media/press-releases (accessed on 25 May 2025). Additional regulatory and safety information is available through national and international health authorities and immunization advisory bodies.

## Figures and Tables

**Table 1:** Overview of VLA1553 clinical trials included in this manuscript.

Trial	Registration Number	Phase	Country	CHIKV Endemic?	Year	Design	Total Number of Participants	Number of Participants in Safety Population	Reference
VLA1553-101	NCT03382964	1	USA	No	2018–2019	Randomized, single-blind, multicenter, dose-escalation trial of three VLA1553 dose levels (low dose: 3.2 × 10^3^ TCID_50_; medium dose: 3.2 × 10^4^ TCID_50_; high dose: 3.2 × 10^5^ TCID_50_)	120 (31 low-dose, 30 medium-dose, 59 high-dose)	120	[31]
VLA1553-301	NCT04546724	3	USA	No	2020–2021	Randomized, double-blind, multicenter trial in two groups, VLA1553 and placebo in a 3:1 randomization	4128 (3093 VLA1553, 1035 placebo)	4115	[32]
VLA1553-302	NCT04786444	3	USA	No	2021	Prospective, randomized, double-blind, multicenter lot-to-lot consistency trial using 3 VLA1553 lots	409 (136 lot 1, 137 lot 2, 136 lot 3)	408	[33]

CHIKV, chikungunya virus; TCID_50_, 50% tissue culture infection dose.

**Table 2:** Adverse events (reported in VLA1553-101, VLA1553-301, and VLA1553-302) included/excluded based on a medical review of the definition of broad-definition AESIs by System Organ Class and Preferred Term.

System Organ Class (SOC)	Preferred Terms (PTs) Included/Excluded
General disorders and administration site conditions	All reported AEs in this SOC except injection site erythema, injection site induration, injection site pain, injection site swelling, vaccination site pain, tenderness, nodule ^a^
Musculoskeletal and connective tissue disorders	Arthralgia, arthritis, polyarthritis, polyarthralgia, back pain, myalgia
Nervous system disorders	All reported AEs in this SOC except postural dizziness, sciatica ^b^
Cardiac disorders	All reported AEs in this SOC
Skin and subcutaneous tissue disorders	All reported AEs in this SOC except contact dermatitis, eczema, sensitive skin ^c^
Blood and lymphatic system disorders	All reported AEs in this SOC except leukopenia, lymphocytosis, lymphopenia, neutropenia ^d^
Eye disorders	Optic neuritis, retinitis, and uveitis

AE, adverse event; AESI, adverse event of special interest; PT, preferred term; SOC, system organ class. To fulfill the definition of a broad-definition AESI, any of the symptoms presented in this table occurred together with fever within 30 days post-vaccination, regardless of the order of their onset or their duration. ^a^ Injection site erythema, injection site induration, injection site pain, injection site swelling, vaccination site pain, and tenderness were excluded as these symptoms were local reactions that occurred after vaccination. The PT of nodule was excluded since the symptom is not considered to be a CHIKV-related symptom. ^b^ Postural dizziness and sciatica were excluded since both were not considered to be CHIKV-related symptoms. Dizziness can occur during an acute CHIKV infection but is not specifically related to posture (note: the PT dizziness was included in the definition of broad-definition AESIs); sciatica was excluded since this symptom is normally caused by injury to or pressure on the sciatic nerve which most often occurs when a herniated disk or an overgrowth of bone puts pressure on part of the nerve. ^c^ Contact dermatitis, eczema, and sensitive skin were excluded since they were not considered to be CHIKV-related symptoms; contact dermatitis is normally caused by direct contact with a substance or an allergic reaction to it; eczema is usually atopic dermatitis that is chronic and needs to be distinguished from temporal rash, which can be present during a viral infection (note: the PTs of rash and erythematous rash were included in the definition of broad-definition AESIs); the PT of sensitive skin is an unspecific term, which can be attributed to a number of recognized medical causes including dermatitis, contact urticaria, rosacea, dry skin, and others; therefore, sensitive skin was not considered a symptom related to CHIKV. ^d^ Leukopenia, lymphopenia, and neutropenia are associated with acute CHIKV infection; however, they were not included in the Sponsor’s AESI definition: they are not considered a symptom of CHIKV disease, which can manifest as any of the clinical forms defined in the AESI definition; rather, they are laboratory abnormalities for CHIKV infection, which were assessed as such in VLA1553 clinical studies (i.e., evaluation of laboratory findings in hematology parameters after vaccination). Moreover, laboratory parameters were not systematically evaluated in VLA1553-301 and VLA1553-302. Including these PTs here would therefore have resulted in an unsystematic broad-definition AESI assessment. Lymphocytosis was excluded for the same reasons and since it was not considered to be a CHIKV-related symptom.

**Table 3:** Summary of episodes of prolonged arthralgia (as part of a broad-definition AESI or solicited arthralgia) for VLA1553 and placebo in VLA1553-301 trial (safety population).

Group	Age/Race/Sex	Broad-Definition AESI Case?	Onset (Day)	Duration (Days)	Severity (By Investigator)	Outcome	Causality	Relevant Medical History
Prolonged arthralgia ≥ 30 days to <3 months duration								
VLA1553	59/B/F	No	1	39	Moderate	Recovered/resolved	Probable	Osteoarthritis (ongoing)
VLA1553	42/B/M ^a^	No	6	41	Mild	Recovered/resolved	Possible	None
VLA1553	46/W/M	Yes	4	48 ^b^	Mild	Recovering/resolving	Probable	Chron’s disease (ongoing)
Placebo	48/W/M	No	4	63	Moderate	Recovered/resolved	Probable	Obese (ongoing)
Prolonged arthralgia > 3 months duration								
VLA1553	61/W/F ^c^	No	7	119	Mild	Recovered/resolved	Unlikely	Blood fibrinogen increased (ongoing)
VLA1553	40/B/M	No	5	124	Severe	Recovered/resolved	Probable	Osteoarthritis bilateral knees, (ongoing) Oedema peripheral (ongoing)
VLA1553	39/W/M ^d^	Yes	7	143 ^e^	Moderate	Not recovered/not resolved	Not related	Left wrist fracture (resolved)
VLA1553	61/W/F	Yes	15 ^f^	154 ^g^	Mild	Not recovered/not resolved	Unlikely	None
VLA1553	44/W/M ^h^	Yes	8	162 ^g^	Mild	Not recovered/not resolved	Not related	None
VLA1553	49/W/F	No	6	165 ^g^	Moderate	Not recovered/not resolved	Possible	Osteoarthritis (resolved)
VLA1553	62/W/F	No	2	166 ^g^	Moderate	Recovering/resolving	Possible	Cervical spinal stenosis (ongoing)
VLA1553	30/W/F	No	7	177	Mild	Recovered/resolved	Possible	None
Placebo	42/W/M	No	2	180 ^g^	Mild	Recovering/resolving	Possible	Back pain (ongoing) Lumbar spinal stenosis (ongoing) Neck pain (ongoing)
VLA1553	50/W/F ^i^	Yes	2	182 ^g^	Moderate	Not recovered/not resolved	Probable	Foot fracture (resolved) Obese (ongoing) Back pain (resolved) HLA-B27 positive

AE, adverse event; AESI, adverse event of special interest; B, Black; F, female; HLA, human leukocyte antigen; M, male; W, White. ^a^ Arthralgia reported as right shoulder pain. ^b^ Ongoing at time of withdrawal. Event duration was calculated with the date the participant withdrew from trial. ^c^ Arthralgia reported as right hip pain. ^d^ Arthralgia reported as arthralgia in the left hand. ^e^ Ongoing at time of loss to follow-up. Event duration was calculated with date the participant was declared as lost to follow-up. ^f^ Unsolicited arthralgia (identified as part of broad-definition AESI). ^g^ Ongoing at end of trial. Event duration was calculated with end of trial date. ^h^ Arthralgia reported as lateral collateral ligament pain in the right knee. ^i^ Arthralgia reported as polyarthralgia and nodular swelling of joints in fingers and foot.

**Table 4:** Summary of episodes of prolonged myalgia (as part of a broad-definition AESI or solicited myalgia) for VLA1553 and placebo in VLA1553-301 trial (safety population).

Group	Age/Race/Sex	Broad-Definition AESI Case?	Onset (Day)	Duration (Days)	Severity (By Investigator)	Outcome	Causality	Relevant Medical History
Prolonged myalgia ≥ 30 days to <3 months duration								
VLA1553	58/W/F	No	2	30	Mild ^a^	Recovered/resolved	Probable	Fibromyalgia (ongoing) Back pain (ongoing) Type 2 diabetes mellitus (ongoing)
VLA1553	59/B/F	No	1	39	Mild	Recovered/resolved	Probable	Osteoarthritis (ongoing)
VLA1553	30/O/M	Yes	4	42 ^b^	Mild	Recovering/resolving	Possible	None
VLA1553	46/W/M	Yes	4	48 ^b^	Mild	Not recovered/not resolved	Possible	Hypercholesterolemia (ongoing) ^c^
VLA1553	54/W/M	No	5	62	Mild	Recovered/resolved	Probable	None
VLA1553	62/I/M	No	5	75	Mild	Recovered/resolved	Probable	Hypercholesterolemia (ongoing) ^c^
Prolonged myalgia > 3 months duration								
VLA1553	38/W/M ^d^	Yes	30 ^e^	142 ^f^	Mild	Not recovered/not resolved	Not related	Obese (ongoing) Spinal osteoarthritis (ongoing) Myalgia (ongoing) Type 2 diabetes mellitus (ongoing)
VLA1553	57/W/F ^g^	No	6	194 ^f^	Mild	Not recovered/not resolved	Unlikely	Type 2 diabetes mellitus (ongoing)

AE, adverse event; AESI, adverse event of special interest; B, Black; F, female; I, American Indian or Alaska native; M, male; O, other; W, White. ^a^ Myalgia reported as severe by the participant but the investigator considered it mild given the participant’s history of fibromyalgia and very low pain tolerance. ^b^ Ongoing at time of withdrawal. Event duration was calculated with the date the participant withdrew from trial. ^c^ Ongoing therapy with statins. ^d^ Myalgia reported as worsening of intermittent right trapezius pain. ^e^ Unsolicited myalgia (identified as part of broad-definition AESIs). ^f^ Ongoing at end of trial. Event duration was calculated with end of trial date. ^g^ Myalgia reported as mild left occipital muscle tenderness.

**Table 5:** Summary of episodes of osteoarthritis and spinal osteoarthritis for VLA1553 and placebo in VLA1553-301 (safety population).

Adverse Event	Age/Race/Gender	BMI (kg/m^2^)	Onset (Day)	Duration (Days)	Reported Verbatim	Osteoarthritis Medical History?	Solicited Systemic Adverse Events (Start Day-End Day), Severity, Causality)
VLA1553							
Osteoarthritis	62/W/F	34.7	153	1	Osteoarthritis in the knees	Yes (ongoing)	Nausea (4–4), mild, possible Headache (6–8), mild, probable
	54/W/F	35.2	17 ^a^	4	Acute right knee osteoarthritis	Yes (ongoing)	None
	44/W/F	28.4	24 ^a^	4	Right hip pain worsening secondary to osteoarthritis	Yes (ongoing)	None
	50/B/M	25.7	10 ^a^	19	Worsening of osteoarthritis	Yes (ongoing)	Nausea (3–3), mild, possible
	60/W/M	25.6	57	19	Worsening of osteoarthritis bilateral hands	Yes (ongoing)	None
	81/W/F	29.6	149	21 ^b^	Worsening of osteoarthritis right shoulder	Yes (ongoing)	None
	60/W/F	42.4	20 ^a^	27	Exacerbation of osteoarthritis bilateral knees	Yes (ongoing)	Myalgia (1–2), mild, possible
	48/W/F	23.4	139	37 ^b^	Degenerative osteoarthritis	No	Myalgia (3–7), mild, possible
	55/W/M	35.9	66	103 ^b^	Early onset osteoarthritis (legs)	No	None
	55/W/F	35.6	49	121 ^b^	Worsening of osteoarthritis bilateral hips	Yes (ongoing)	Myalgia (4–6), mild, possible
	76/W/F	38.1	8 ^a^	174 ^b^	Chronic pain osteoarthritis (worsening)	Yes (ongoing)	Vomiting (9–10), mild, possible Fatigue (9–10), mild, possible Pyrexia (4–5), mild, probable Arthralgia (4–10), mild, probable Myalgia (9–10), mild, possible Headache (4–10), mild, probable
Spinal osteo arthritis	38/W/M	48.1	30	142 ^b^	Worsening of osteoarthritis cervical spine	Yes (ongoing)	Nausea (1–1), mild, probable Fatigue (1–10), mild probable Pyrexia (7–8), severe, probable Myalgia (7–22), moderate, probable Headache (7–9), mild, probable
Placebo							
Osteoarthritis	62/W/M	29.7	74	114 ^b^	Osteoarthritis pain to bilateral hands	Yes (ongoing)	None
	56/B/F	39.5	27 ^a^	158 ^b^	Exacerbation of osteoarthritis	Yes (ongoing)	Myalgia (2–2), mild, probable

^a^ Worsening of osteoarthritis that started within 4 weeks post-vaccination. ^b^ Ongoing at end of trial—event duration was calculated with end of trial date.

**Table 6:** Symptoms of broad-definition AESIs for participants in the safety population for VLA1553 and placebo in VLA1553-301 trial (safety population).

	VLA1553 (*N* = 3082)		Placebo (*N* = 1033)	
	*n* (%) Obs		*n* (%) Obs	
	Overall	Severe	Overall	Severe
Any broad-definition AESI symptom	361 (11.7) 1389	48 (1.6) 52	6 (0.6) 20	0
Pyrexia	361 (11.7) 361	39 (1.3) 39	6 (0.6) 6	0
Headache	280 (9.1) 282	1 (<0.1) 1	5 (0.5) 5	0
Fatigue	264 (8.6) 265	2 (0.1) 2	5 (0.5) 5	0
Myalgia	215 (7.0) 217	3 (0.1) 3	1 (0.1) 1	0
Arthralgia	159 (5.2) 162	5 (0.2) 5	2 (0.2) 2	0
Chills	29 (0.9) 29	0	0	-
Rash	22 (0.7) 24	0	0	-
Back pain	13 (0.4) 13	1 (<0.1) 1	0	-
Lymphadenopathy	9 (0.3) 10	0	0	-
Dizziness	6 (0.2) 6	0	0	-
Pain	4 (0.1) 4	0	0	-
Paresthesia	3 (0.1) 3	0	0	-
Hyperhidrosis	2 (0.1) 2	0	0	-
Oedema peripheral	2 (0.1) 2	0	0	-
Asthenia	1 (<0.1) 1	0	0	-
Ataxia	1 (<0.1) 1	0	0	-
Atrial fibrillation	1 (<0.1) 1	1 (<0.1) 1	0	-
Chest pain	0	-	1 (0.1) 1	0
Feeling abnormal	1 (<0.1) 1	0	0	-
Hypoaesthesia	1 (<0.1) 1	0	0	-
Influenza-like illness	1 (<0.1) 1	0	0	-
Neuropathy peripheral	1 (<0.1) 1	0	0	-
Rash erythematous	1 (<0.1) 1	0	0	-
Syncope	1 (<0.1) 1	0	0	-

AESI, adverse event of special interest; *n*, number of participants with broad-definition AESI symptom; *N*, number of participants in group; %, percentage based on *N*; Obs, number of events.

**Table 7:** Summary of prolonged (symptom lasting ≥ 30 days) broad-definition AESIs for VLA1553 and placebo in VLA1553-301 (safety population).

	VLA1553 (*N* = 3082)	Placebo (*N* = 1033)
	*n* (%) Obs	*n* (%) Obs
Any prolonged broad-definition AESI symptom	14 (0.5) 21	0
*Severe*	*1 (<0.1) 1*	*-*
Arthralgia	5 (0.2) 6	0
*Severe*	*0*	*-*
Fatigue	3 (0.1) 3	0
*Severe*	*0*	*-*
Headache	3 (0.1) 3	0
*Severe*	*0*	*-*
Myalgia	3 (0.1) 3	0
*Severe*	*0*	*-*
Lymphadenopathy	2 (0.1) 2	0
*Severe*	*0*	*-*
Back pain	1 (<0.1) 1	0
*Severe*	*1 (<0.1) 1*	*-*
Oedema peripheral	1 (<0.1) 1	0
*Severe*	*0*	*-*
Fever	1 (<0.1) 1	0
*Severe*	*0*	*-*
Rash erythematous	1 (<0.1) 1	0
*Severe*	*0*	*-*

AESI, adverse event of special interest; *n*, number of participants with prolonged broad-definition AESI symptom; *N*, number of participants in group; %, percentage based on *N*; Obs, number of events.

**Table 8:** Summary of broad-definition AESIs following VLA1553 from pooled analysis (safety population).

	VLA1553 (Pooled Analysis) ^a^		
	Any Broad Definition AESI *n* (%)	Any Severe Broad-Definition AESI *n* (%)	Any Related Broad-Definition AESI *n* (%)
Age group			
18–45 years (*N* = 2084)	283 (13.6)	49 (2.4)	270 (13.0)
46–64 years (*N* = 1180)	116 (9.8)	15 (1.3)	113 (9.6)
65–74 years (*N* = 287)	32 (11.1)	1 (0.3)	30 (10.5)
75–84 years (*N* = 54)	4 (7.4)	0	4 (7.4)
≥85 years (*N* = 5)	1 (20.0)	0	1 (20.0)
Sex			
Female (*N* = 1919)	182 (9.5)	21 (1.1)	174 (9.1)
Male (*N* = 1691)	254 (15.0)	44 (2.6)	244 (14.4)
Ethnicity			
Hispanic or Latino (*N* = 608)	67 (11.0)	14 (2.3)	65 (10.7)
Not Hispanic or Latino (*N* = 2961)	365 (12.3)	50 (1.7)	349 (11.8)
Race			
American Indian or Alaska Native (*N* = 33)	3 (9.1)	0	3 (9.1)
Asian (*N* = 74)	11 (14.9)	2 (2.7)	10 (13.5)
Black or African American (*N* = 530)	48 (9.1)	8 (1.5)	48 (9.1)
Native Hawaiian or Other Pacific Islander (*N* = 14)	1 (7.1)	0	1 (7.1)
White (*N* = 2867)	362 (12.6)	54 (1.9)	345 (12.0)
Other (*N* = 92)	11 (12.0)	1 (1.1)	11 (12.0)
Body mass index			
<25 kg/m^2^ (*N* = 884)	114 (12.9)	11 (1.2)	107 (12.1)
≥25 kg/m^2^ to <30 kg/m^2^ (*N* = 1126)	136 (12.1)	23 (2.0)	131 (11.6)
≥30 kg/m^2^ to <35 kg/m^2^ (*N* = 833)	90 (10.8)	17 (2.0)	88 (10.6)
≥35 kg/m^2^ (*N* = 763)	96 (12.6)	14 (1.8)	92 (12.1)

^a^ Pooled analysis used all data from trials VLA1553-101, VLA1553-301, and VLA1553-302. AESI, adverse event of special interest; *N*, number of participants in group; %, percentage based on *N*.

## Data Availability

Data are available from the corresponding author upon reasonable request.

## Supplementary Materials

The following supporting information can be downloaded at: https://www.mdpi.com/article/10.3390/vaccines13060576/s1, Figure S1: Incidence of solicited systemic reactions by age for VLA1553 and placebo in VLA1553-301 (safety population); Figure S2: Incidence of solicited arthralgia and fever by duration for VLA1553 and placebo in VLA1553-301 (safety population); Figure S3: Symptoms of broad-definition AESIs occurring at an incidence of >5% (all severities) for VLA1553 recipients with broad-definition AESIs in VLA1553-301 trial (safety population); Table S1: Summary of episodes of prolonged arthralgia (as part of a broad-definition AESI or solicited arthralgia) for VLA1553 and placebo in VLA1553-301 trial (safety population); Table S2: Summary of episodes of prolonged myalgia (as part of a broad-definition AESI or solicited myalgia) for VLA1553 and placebo in VLA1553-301 trial (safety population).
