# Supplementary material for: Comprehensive Assessment of Reactogenicity and Safety of the Live-Attenuated Chikungunya Vaccine (IXCHIQ®)
Source: Vaccines (Basel). 2025 May 28;13(6):576. doi: 10.3390/vaccines13060576 (PMC12197765; doi:10.3390/vaccines13060576)

SUPPLEMENTARY APPENDIX

Supplementary Table S1 Summary of episodes of prolonged arthralgia (as part of a broad definition AESI or solicited arthralgia) for VLA1553 and placebo in trial VLA1553-301 (safety population)

Group	Age/ Race/ Sex	Broad definition AESI case?	Onset (Day)	Duration (Days)	Severity (by Investigator)	Outcome	Causality	Completed trial?	Other symptoms (start day-end day), severity, causality	Relevant medical history
Prolonged arthralgia ≥30 days to <3 months duration										
VLA1553	59/B/F	No	1	39	Moderate	Recovered/ resolved	Probable	Yes	*Solicited systemic AEs:*<br>Fatigue (1-39), mild, probable<br>Myalgia (1-39), mild, probable<br>Headache (1-39), mild, probable	Osteoarthritis (ongoing)
VLA1553	42/B/M[a]	No	6	41	Mild	Recovered/ resolved	Possible	No (participant withdrew [Day 149])	None	None
VLA1553	46/W/M	Yes	4	48[b]	Mild	Recovering/ resolving	Probable	No (participant withdrew [Day 51])	*Broad definition AESI symptoms (solicited):*<br>Fatigue (6-8), mild, possible<br>Fever (2-6), moderate, probable<br>Myalgia (4-51)[b], mild, possible<br>Headache (4-7), mild, possible<br>*Broad definition AESI symptoms (unsolicited):*<br>Back pain (2-51)[b], mild[c], probable	Chron's disease (ongoing)
Placebo	48/W/M	No	4	63	Moderate	Recovered/ resolved	Probable	Yes	*Solicited systemic AEs:*<br>Myalgia (2-4), mild, probable	Obese (ongoing)
Prolonged arthralgia >3 months duration										
VLA1553	61/W/F[d]	No	7	119	Mild	Recovered/ resolved	Unlikely	Yes	*Solicited systemic AEs:*<br>Nausea (11-12), mild, unlikely<br>Headache (4-8), mild, unlikely	Blood fibrinogen increased (ongoing)
VLA1553	40/B/M	No	5	124	Severe	Recovered/ resolved	Probable	Yes	*Solicited systemic AEs:*<br>Nausea (5-7), moderate, probable<br>Fatigue (5-16), moderate, probable<br>Headache (5-7), moderate, probable<br>Rash (5-168), mild, not related	Osteoarthritis bilateral knees, (ongoing)<br>Oedema peripheral (ongoing)

Group	Age/ Race/ Sex	Broad definition AESI case?	Onset (Day)	Duration (Days)	Severity (by Investigator)	Outcome	Causality	Completed trial?	Other symptoms (start day-end day), severity, causality	Relevant medical history
VLA1553	39/W/M[e]	Yes	7	143[f]	Moderate	Not recovered/ not resolved	Not related	No (lost to follow-up [Day 149])	*Broad definition AESI symptoms (solicited):*<br>Fatigue (1-1), mild, possible<br>Fever (4-7), moderate, possible<br>Myalgia (4-17), moderate, possible<br>Headache (1-7), moderate, possible<br>*Broad definition AESI symptoms (unsolicited):*<br>Chills (4-7), moderate, possible	Left wrist fracture (resolved)
VLA1553	61/W/F	Yes	15[g]	154[h]	Mild	Not recovered/ not resolved	Unlikely	Yes	*Broad definition AESI symptoms (solicited):*<br>Fever (5-5), mild, probable<br>Headache (4-6), mild, possible<br>Arthralgia, (5-6), mild, probable	None
VLA1553	44/W/M[i]	Yes	8	162[h]	Mild	Not recovered/ not resolved	Not related	Yes	*Broad definition AESI symptoms (solicited):*<br>Fatigue (4-5), mild, probable<br>Fever (4-5), mild, probable<br>Myalgia (4-5), mild, probable<br>Headache (4-5), mild, probable	None
VLA1553	49/W/F	No	6	165[h]	Moderate	Not recovered/ not resolved	Possible	Yes	*Solicited systemic AEs:*<br>Nausea (6-6), mild, possible<br>Fatigue (9-10), mild, possible<br>Myalgia (6-8), moderate, possible	Osteoarthritis (resolved)
VLA1553	62/W/F	No	2	166[h]	Moderate	Recovering/ resolving	Possible	Yes	*Solicited systemic AEs:*<br>Nausea (7-11), mild, probable<br>Fatigue (7-11), mild, probable<br>Myalgia (2-2), mild, probable	Cervical spinal stenosis (ongoing)
VLA1553	30/W/F	No	7	177	Mild	Recovered/ resolved	Possible	Yes	*Solicited systemic AEs:*<br>Myalgia (4-10), mild, possible<br>Headache (1-8) mild, possible	None
Placebo	42/W/M	No	2	180[h]	Mild	Recovering/ resolving	Possible	Yes	*Solicited systemic AEs:*<br>Nausea (5-11), moderate, possible<br>Fatigue (1-3), moderate, possible<br>Headache (11-11), mild, unlikely	Back pain (ongoing)<br>Lumbar spinal stenosis (ongoing)

Group	Age/ Race/ Sex	Broad definition AESI case?	Onset (Day)	Duration (Days)	Severity (by Investigator)	Outcome	Causality	Completed trial?	Other symptoms (start day-end day), severity, causality	Relevant medical history
										Neck pain (ongoing)
VLA1553	50/W/F[j]	Yes	2	182[h]	Moderate	Not recovered/ not resolved	Probable	Yes	*Broad definition AESI symptoms (solicited):*<br>Nausea (5-5), mild, unlikely<br>Fatigue (6-6), mild, possible<br>Fever (5-6), mild, possible<br>Myalgia (2-5), mild, possible<br>Headache (2-14), mild, possible<br>*Broad definition AESI symptoms (unsolicited):*<br>Oedema peripheral (11-183), mild, probable	Foot fracture (resolved)<br>Obese (ongoing)<br>Back pain (resolved)<br>HLA-B27 positive

AE, adverse event; AESI, adverse event of special interest; B, Black; F, female; HLA, human leukocyte antigen; M, male; W, White.

[a]Arthralgia reported as right shoulder pain.

[b]Ongoing at time of withdrawal. Event duration was calculated with the date the participant withdrew from trial.

[c]Event was documented as mild and was later on described as severe pain in the centre of the back by the participant.

[d]Arthralgia reported as right hip pain.

[e]Arthralgia reported as arthralgia in left hand.

[f]Ongoing at time of lost to follow-up. Event duration was calculated with date the participant was declared as lost to follow-up.

[g]Unsolicited arthralgia (identified as part of broad definition AESI).

[h]Ongoing at end of trial. Event duration was calculated with end of trial date.

[i]Arthralgia reported as lateral collateral ligament pain in the right knee.

[j]Arthralgia reported as polyarthralgia and nodular swelling of joints in fingers and foot.

Supplementary Table S2 Summary of episodes of prolonged myalgia (as part of a broad definition AESI or solicited myalgia) for VLA1553 and placebo in trial VLA1553-301 (safety population)

Group	Age/ Race/ Sex	Broad definition AESI case?	Onset (Day)	Duration (Days)	Severity (by Investigator)	Outcome	Causality	Completed trial?	Other symptoms (start day-end day), severity, causality	Relevant medical history
Prolonged myalgia ≥30 days to <3 months duration										
VLA1553	58/W/F	No	2	30	Mild[a]	Recovered/ resolved	Probable	Yes	*Solicited systemic AEs:*<br>Arthralgia (3-3), mild, probable<br>Headache (2-7), mild, probable	Fibromyalgia (ongoing)<br>Back pain (ongoing)<br>Type 2 diabetes mellitus (ongoing)
VLA1553	59/B/F	No	1	39	Mild	Recovered/ resolved	Probable	Yes	*Solicited systemic AEs:*<br>Fatigue (1-39), mild, probable<br>Arthralgia (1-39), moderate, probable<br>Headache (1-39), mild, probable	Osteoarthritis (ongoing)
VLA1553	30/O/M	Yes	4	42[b]	Mild	Recovering/ resolving	Possible	No (participant withdrew [Day 45])	*Broad definition AESI symptoms (solicited):*<br>Fatigue (4-5), mild, possible<br>Fever (4-4), mild, probable<br>Headache (4-5), moderate, probable	None
VLA1553	46/W/M	Yes	4	48[b]	Mild	Not recovered/ not resolved	Possible	No (participant withdrew [Day 51])	*Broad definition AESI symptoms (solicited):*<br>Fatigue (6-8), mild, possible<br>Fever (2-6), moderate, probable<br>Arthralgia (4-51)[b], mild, probable<br>Headache (4-7), mild, possible<br>*Broad definition AESI symptoms (unsolicited):*<br>Back pain (2-51)[b], mild[c], probable	Hypercholesterol-aemia (ongoing)[d]
VLA1553	54/W/M	No	5	62	Mild	Recovered/ resolved	Probable	Yes	*Solicited systemic AEs:*<br>Arthralgia (5-9), mild, probable	None

Group	Age/ Race/ Sex	Broad definition AESI case?	Onset (Day)	Duration (Days)	Severity (by Investigator)	Outcome	Causality	Completed trial?	Other symptoms (start day-end day), severity, causality	Relevant medical history
VLA1553	62/I/M	No	5	75	Mild	Recovered/ resolved	Probable	Yes	*Solicited systemic AEs:*<br>Nausea (5-5), mild, probable<br>Fatigue (5-9), mild, probable<br>Arthralgia (8-8), mild, probable<br>Headache (5-7), mild, probable	Hypercholesterol-aemia (ongoing)[d]
Prolonged myalgia >3 months duration										
VLA1553	38/W/M[e]	Yes	30[f]	142[g]	Mild	Not recovered/ not resolved	Not related	Yes	*Broad definition AESI symptoms (solicited):*<br>Nausea (1-1), mild, probable<br>Fatigue (1-10), mild, probable<br>Fever (7-8), severe, probable<br>Headache (7-9), mild, probable<br>Myalgia (7-22), moderate, probable<br>*Broad definition AESI symptoms (unsolicited):*<br>Back pain (7-8), mild, probable	Obese (ongoing)<br>Spinal osteoarthritis (ongoing)<br>Myalgia (ongoing)<br>Type 2 diabetes mellitus (ongoing)
VLA1553	57/W/F[h]	No	6	194[g]	Mild	Not recovered/ not resolved	Unlikely	Yes	*Solicited systemic AEs:*<br>Fatigue (1-7), mild, probable<br>Arthralgia (8-9), mild, not related	Type 2 diabetes mellitus (ongoing)

AE, adverse event; AESI, adverse event of special interest; B, Black; F, female; I, American Indian or Alaska native; M, male; O, other; W, White.

[a]Myalgia reported as severe by the participant but the investigator considered it mild given the participant's history of fibromyalgia and very low pain tolerance.

[b]Ongoing at time of withdrawal. Event duration was calculated with the date the participant withdrew from trial.
[c]Event was documented as mild and was later on described as severe pain in the centre of the back by the participant.
[d]Ongoing therapy with statins.

[e]Myalgia reported as worsening of intermittent right trapezius pain.

[f]Unsolicited myalgia (identified as part of broad definition AESI).

[g]Ongoing at end of trial. Event duration was calculated with end of trial date.

[h]Myalgia reported as mild left occipital muscle tenderness.

Supplementary Figure S1 Incidence of solicited systemic reactions by age for VLA1553 and placebo in VLA1553-301 (safety population)

A. Fever

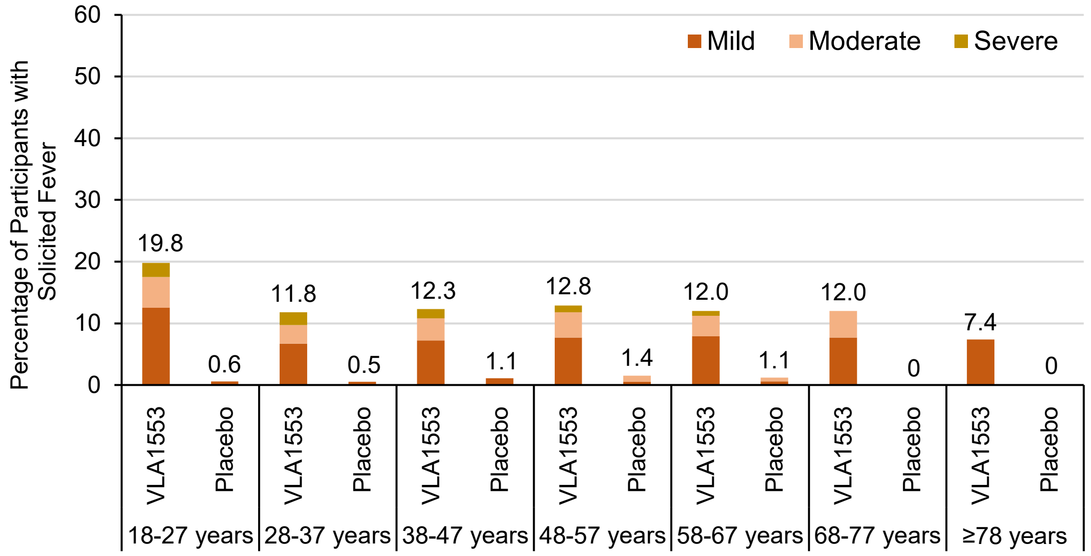

Note: The number of participants (n) included in each age group was as follows: in the VLA1553 group: 18-27 years (n=519), 28-37 years (n=569), 38-47 years (n=586), 48-57 years (n=664), 58-67 years (n=509), 68-77 years (n=208), ≥78 years (n=27); in the placebo group:18-27 years (n=168), 28-37 years (n=210), 38-47 years (n=186), 48-57 years (n=207), 58-67 years (n=177), 68-77 years (n=77), ≥78 years (n=8).

B. Arthralgia

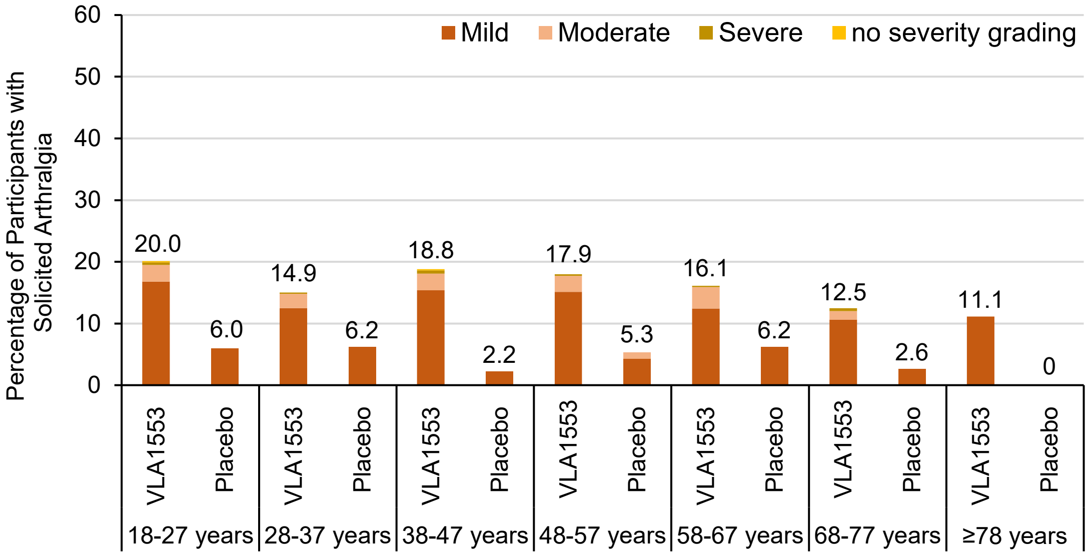

Notes: Diary only adverse events (n=2 in the VLA1553 group) have not had severity captured. The number of participants (n) included in each age group was as follows: in the VLA1553 group: 18-27 years (n=519), 28-37 years (n=569), 38-47 years (n=586), 48-57 years (n=664), 58-67 years (n=509), 68-77 years (n=208), ≥78 years (n=27); in the placebo group:18-27 years

(n=168), 28-37 years (n=210), 38-47 years (n=186), 48-57 years (n=207), 58-67 years (n=177), 68-77 years (n=77), ≥78 years (n=8).

C. Myalgia

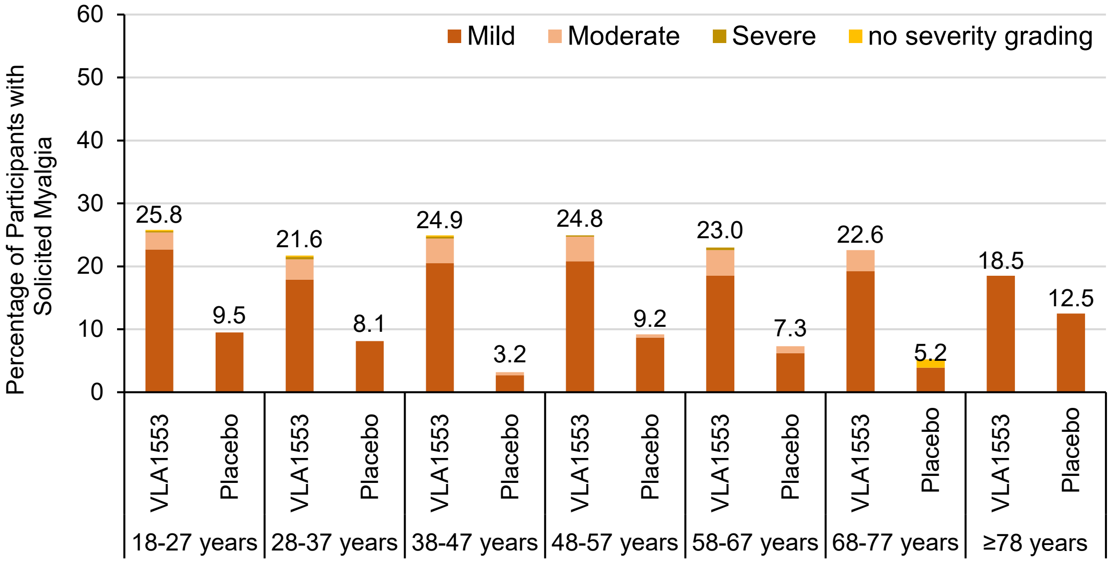

Notes: Diary only adverse events (n=3 in the VLA1553 group, n=1 in the placebo group) have not had severity captured. The number of participants (n) included in each age group was as follows: in the VLA1553 group: 18-27 years (n=519), 28-37 years (n=569), 38-47 years (n=586), 48-57 years (n=664), 58-67 years (n=509), 68-77 years (n=208), ≥78 years (n=27); in the placebo group: 18-27 years (n=168), 28-37 years (n=210), 38-47 years (n=186), 48-57 years (n=207), 58-67 years (n=177), 68-77 years (n=77), ≥78 years (n=8).

D. Fatigue

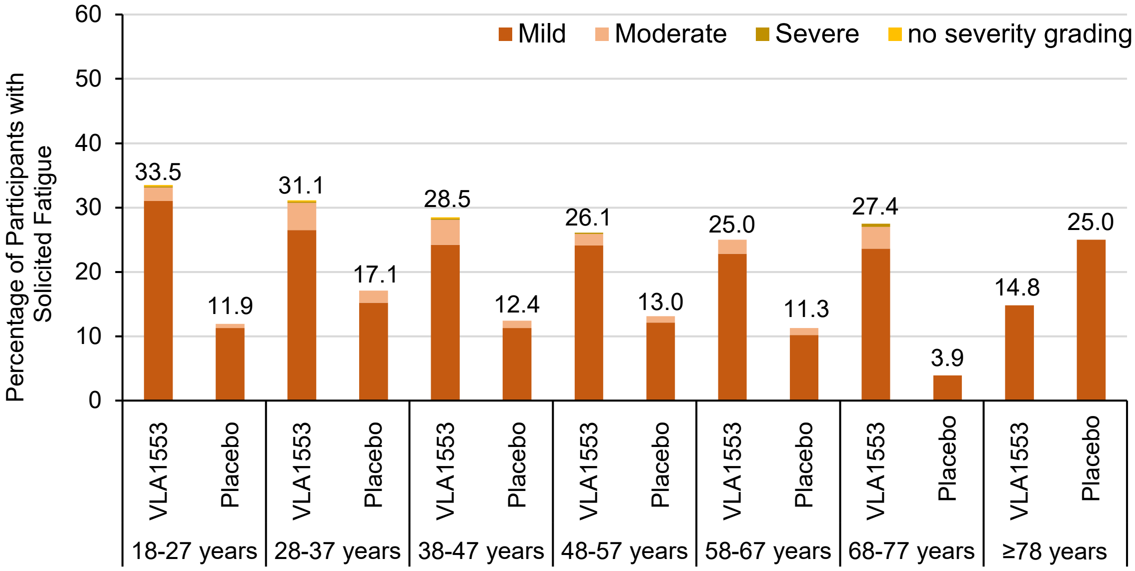

Notes: Diary only adverse events (n=3 in the VLA1553 group) have not had severity captured. The number of participants (n) included in each age group was as follows: in the VLA1553 group: 18-27 years (n=519), 28-37 years (n=569), 38-47 years (n=586), 48-57 years (n=664), 58-67 years (n=509), 68-77 years (n=208), ≥78 years (n=27); in the placebo group: 18-27 years (n=168), 28-37 years (n=210), 38-47 years (n=186), 48-57 years (n=207), 58-67 years (n=177), 68-77 years (n=77), ≥78 years (n=8).

E. Headache

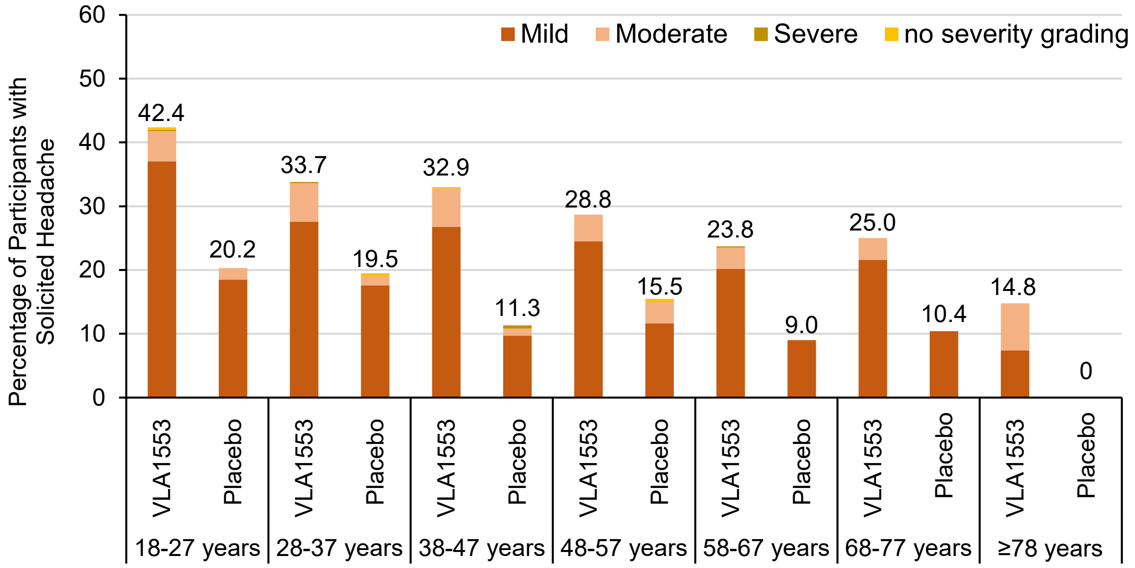

Notes: Diary only adverse events (n=3 in the VLA1553 group, n=2 in the placebo group) have not had severity captured. The number of participants (n) included in each age group was as follows: in the VLA1553 group: 18-27 years (n=519), 28-37 years (n=569), 38-47 years (n=586), 48-57 years (n=664), 58-67 years (n=509), 68-77 years (n=208), ≥78 years (n=27); in the placebo group: 18-27 years (n=168), 28-37 years (n=210), 38-47 years (n=186), 48-57 years (n=207), 58-67 years (n=177), 68-77 years (n=77), ≥78 years (n=8).

Supplementary Figure S2 Incidence of solicited arthralgia and fever by duration for VLA1553 and placebo in VLA1553-301 (safety population)

A. Fever

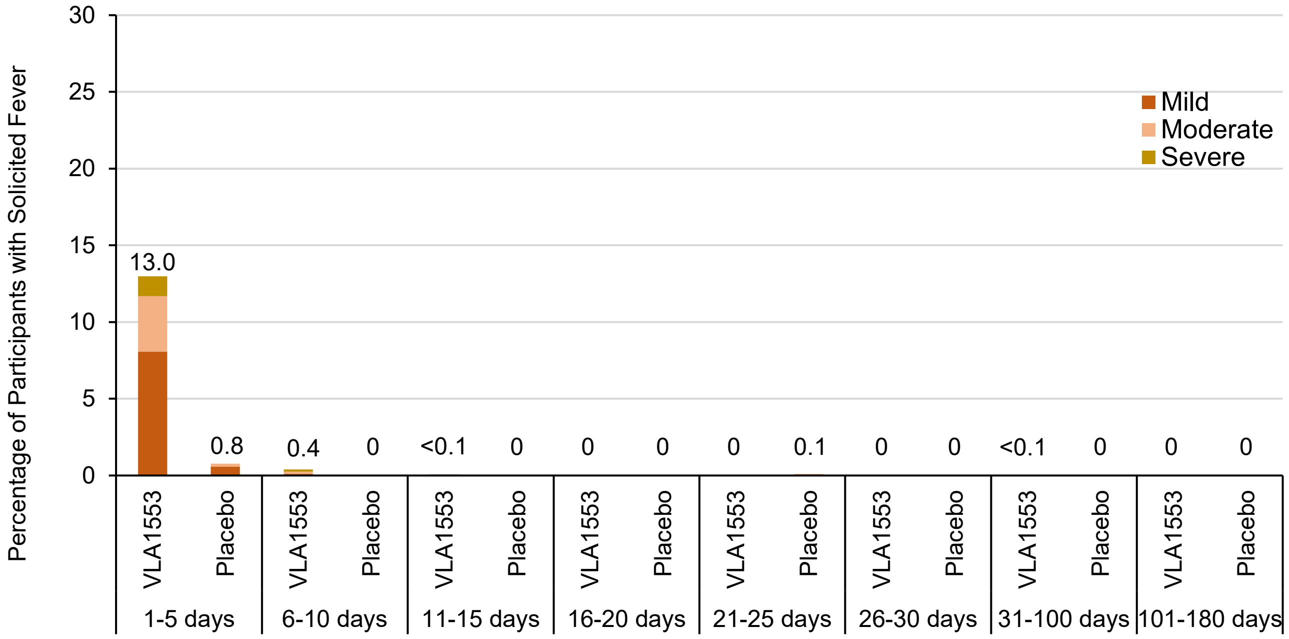

B. Arthralgia

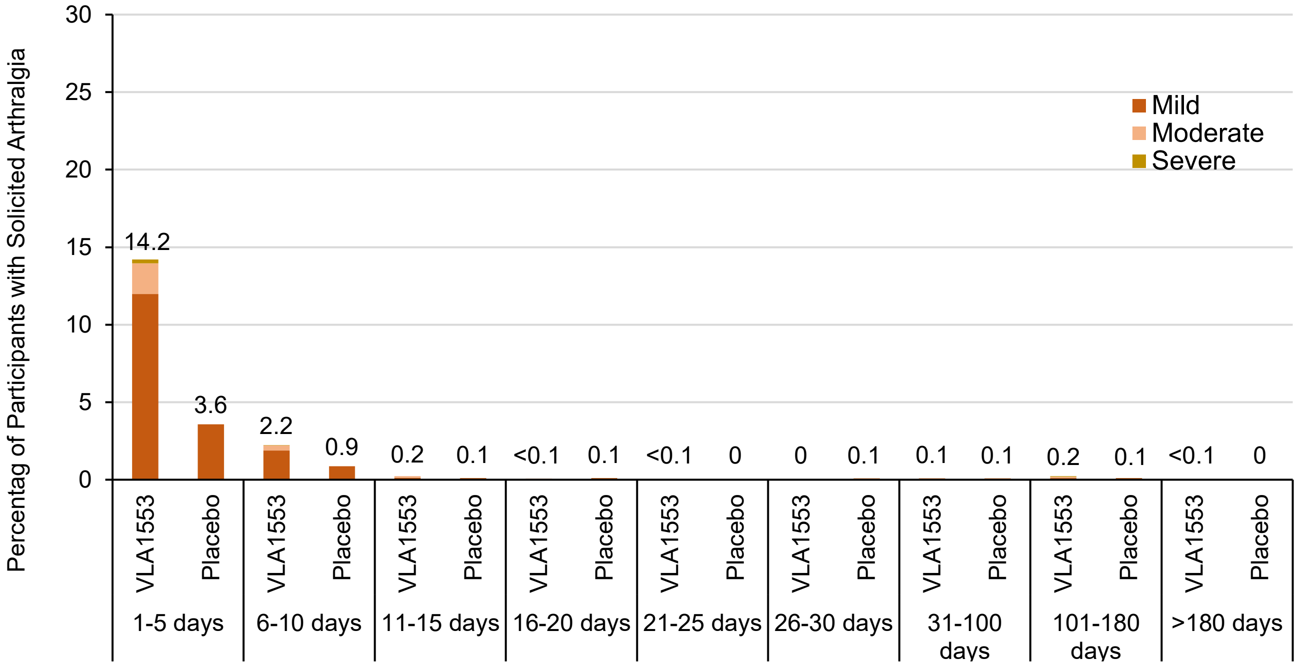

Supplementary Figure S3 Symptoms of broad definition AESI occurring at an incidence of >5% (all severities) for VLA1553 recipients with broad definition AESI in trial VLA1553-301 (safety population)

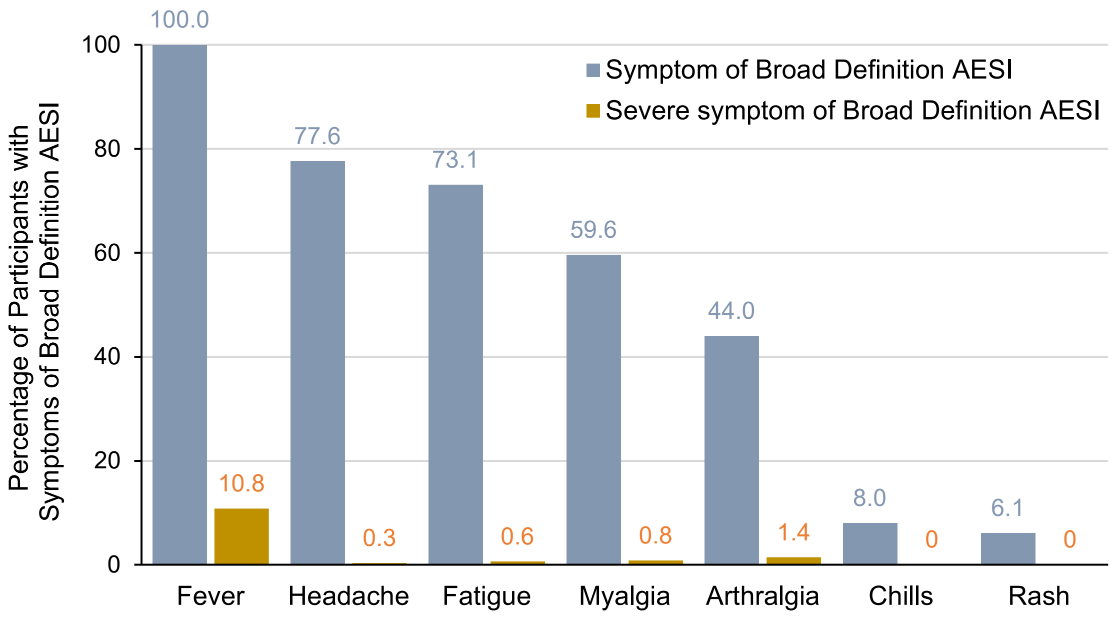

Supplement: Supplementary file 1 [file vaccines-13-00576-s001.zip › vaccines-3589586-supplementary.pdf]
